# Knowledge, Perceptions and Acceptance of Selective Dry Cow Therapy Among Romanian Dairy Farmers and Veterinarians

**DOI:** 10.3390/ani16152401

**Published:** 2026-08-04

**Authors:** Ionela Delia Ut, Daniel Ionut Berean, Liviu Marian Bogdan, Simona Ciupe, Sidonia Gog Bogdan

**Affiliations:** 1 Department of Reproduction, Faculty of Veterinary Medicine, University of Agricultural Sciences and Veterinary Medicine, Calea Manastur 3–5, 400372 Cluj-Napoca, Romania; ionela-delia.ut@student.usamvcluj.ro (I.D.U.); liviu.bogdan@usamvcluj.ro (L.M.B.); simona.ciupe@usamvcluj.ro (S.C.); 2 Department of Surgery, Anesthesiology and Intensive Care, Faculty of Veterinary Medicine, University of Agricultural Sciences and Veterinary Medicine, Calea Manastur 3–5, 400372 Cluj-Napoca, Romania; sidonia.bogdan@usamvcluj.ro

**Keywords:** antimicrobial resistance, antimicrobial stewardship, dairy cattle, mastitis, questionnaire survey

## Abstract

Reducing antimicrobial use in dairy cattle is essential for limiting antimicrobial resistance while maintaining udder health. Selective dry cow therapy (SDCT), in which antibiotics are administered only to cows at increased risk of intramammary infection, has become an important antimicrobial stewardship strategy, yet information regarding its acceptance in Romania is lacking. This cross-sectional study evaluated the knowledge, perceptions, and acceptance of antimicrobial resistance and SDCT among Romanian dairy farmers and veterinarians using an online questionnaire completed by 132 respondents. The questionnaire assessed current dry-off practices, awareness of antimicrobial resistance and selective dry cow therapy, previous implementation, perceived barriers and facilitators, and willingness to adopt or recommend selective dry cow therapy. Overall, 70.5% of farmers had previously heard of antimicrobial resistance, whereas only 27.9% were familiar with the formal concept of SDCT. Administration of antibiotics only to selected cows at dry-off was already the most commonly reported practice among farmers (41.0%) and veterinarians (47.9%), indicating that selective antibiotic use was already being applied despite limited familiarity with the formal SDCT concept. After receiving standardized information describing the strategy, most respondents expressed favorable attitudes and willingness to implement or recommend selective dry cow therapy. Lack of knowledge, limited availability of herd-level diagnostic information, and concerns regarding mastitis were identified as the principal barriers to implementation. Multivariable analysis demonstrated that concern regarding antimicrobial resistance was the only independent predictor of SDCT acceptance among farmers. These findings suggest that strengthening antimicrobial stewardship education, veterinary guidance, standardized protocols, and access to diagnostic tools may facilitate wider adoption of selective dry cow therapy in Romania.

## Simple Summary

Reducing antibiotic use in dairy farming has become an important priority because excessive use contributes to the development of bacteria that no longer respond to treatment. One strategy that helps reduce antibiotic use is selective dry cow therapy, in which only cows at high risk of udder infection receive antibiotics at the end of lactation, while low risk cows do not. Although this approach has been successfully adopted in several countries, little is known about how it is perceived in Romania. This study investigated the knowledge, attitudes, experiences, and willingness of Romanian dairy farmers and veterinarians to use or recommend this strategy through an online questionnaire completed by 132 participants. Most respondents were familiar with the problem of antimicrobial resistance. Although many farmers reported already administering antibiotics only to selected cows at dry-off, relatively few were familiar with the formal concept and terminology of selective dry cow therapy before participating in the survey. After receiving clear information about the strategy most farmers and veterinarians expressed positive attitudes and willingness to adopt or recommend it. Respondents identified lack of information, limited herd health records, and insufficient diagnostic testing as the main barriers to implementation. Farmers who were more concerned about antimicrobial resistance were also more willing to adopt selective dry cow therapy. These findings indicate that improving education, veterinary guidance, and access to diagnostic tools could facilitate the wider implementation of this strategy and contribute to more responsible antibiotic use in Romanian dairy farming.

## 1. Introduction

Antimicrobial resistance (AMR) is currently recognized as one of the greatest threats to global public health, compromising the effective treatment of bacterial infections in both human and veterinary medicine [1]. The extensive use of antimicrobials in food-producing animals, together with their widespread application in human medicine, has been identified as one of the main drivers of AMR development and dissemination [2]. Consequently, AMR has become a major public health concern worldwide and is increasingly recognized not only by the scientific community but also by the general public in many countries [3]. In the dairy sector, antimicrobials are primarily used for the treatment of clinical mastitis (CM) and for dry cow therapy (DCT). Previous studies have estimated that approximately 60% of the total antimicrobial use in dairy cows is associated with mastitis, with nearly two-thirds of this use occurring during DCT [4].

For decades, blanket dry cow therapy (BDCT), consisting of the prophylactic administration of intramammary antibiotics to all cows at dry-off, has represented one of the cornerstones of mastitis control programs [5]. This approach aims both to eliminate existing intramammary infections and to prevent new infections during the dry period [6]. However, the routine administration of antibiotics, including to healthy animals, contributes to unnecessary antimicrobial exposure and increases the risk of antimicrobial resistance development [7]. Consequently, important legislative changes have recently been introduced within the European Union to promote more prudent antimicrobial use in veterinary medicine. Since January 2022, Regulation (EU) 2019/6 has prohibited the routine prophylactic use of antibiotics in food-producing animals, creating an urgent need for sustainable alternatives to blanket dry cow therapy [8].

Selective dry cow therapy (SDCT) has emerged as one such alternative. This strategy involves administering antimicrobials only to cows at increased risk of intramammary infection (IMI), while healthy cows are managed without antibiotics, typically receiving an internal teat sealant (ITS) instead [9]. Selection of cows eligible for antimicrobial treatment is generally based on individual somatic cell count (SCC), CM history, and other indicators of udder health [5]. An effective SDCT protocol should accurately identify cows at high risk of IMI while avoiding unnecessary antimicrobial treatment in healthy animals. However, no universal consensus currently exists regarding the optimal selection criteria for either cows or herds. This variability likely reflects the limited availability of standardized comparative studies, differences in national legislation governing veterinary antimicrobial use, structural differences among dairy industries, variation in pathogen epidemiology, and differences in farmers’ and veterinarians’ attitudes toward dry cow therapy [10,11,12].

Recent evidence indicates that SDCT performs comparably to BDCT regarding the prevention of new IMIs during the dry period, postpartum CM incidence, and SCC during early lactation [13,14,15,16,17,18,19,20,21,22]. Likewise, no significant differences have been reported in milk yield or culling rates during the first 120 days in milk, irrespective of the selection criteria applied, provided that internal teat sealants are used concomitantly [13,15,16,18,19,23]. These findings have supported the widespread implementation of SDCT in several countries, particularly in the Netherlands and the Nordic countries, where substantial reductions in antimicrobial use have already been achieved without compromising udder health [21,24,25,26].

Despite these encouraging results, SDCT implementation remains limited in Romania. Romanian dairy production is characterized by considerable heterogeneity regarding herd size, production systems, milk recording participation, access to bacteriological diagnostics, and herd health management practices [22]. These differences may substantially influence the feasibility of SDCT implementation and suggest that evidence generated in countries where SDCT is already well established cannot be directly extrapolated to Romanian dairy herds. Furthermore, because BDCT has long been considered the standard approach in Romania, transitioning toward SDCT requires substantial changes in routine farm management and decision-making.

Successful implementation of SDCT depends not only on technical and economic factors but also on farmers’ and veterinarians’ knowledge, perceptions, and attitudes toward antimicrobial use (AMU). Although SDCT has been successfully implemented in several countries, its adoption is strongly influenced by behavioural and psychological factors, including attitudes toward udder health, disease prevention, and antimicrobial stewardship [8,11,27,28,29,30,31]. Because BDCT has long been considered the standard approach, transitioning to SDCT requires important changes in routine farm management and decision-making. Farmers who anticipate negative consequences associated with withholding antibiotics are generally less willing to adopt selective treatment. For example, in the Netherlands, farmers expressing negative perceptions regarding SDCT and antimicrobial use were less likely to implement this strategy [30], while in the United Kingdom approximately 55% of dairy farmers reported concerns that SDCT could increase mastitis incidence or even cow mortality [9]. These findings emphasize that successful SDCT implementation depends not only on scientific evidence supporting its efficacy but also on understanding the behavioural factors that influence farmers’ willingness to adopt new management strategies.

In Romania, information regarding the awareness, perceptions, and acceptability of SDCT among both dairy farmers and veterinarians is currently lacking. Likewise, no studies have evaluated stakeholders’ perceptions of antimicrobial resistance or the perceived contribution of the dairy sector to AMR development. Consequently, a systematic evaluation of current knowledge, attitudes, and practices regarding AMU and SDCT implementation is needed. Therefore, the aim of the present study was to evaluate the knowledge, perceptions, and willingness of a sample of Romanian dairy farmers and veterinarians to adopt SDCT. Specifically, the objectives were to: (i) evaluate current dry-off antimicrobial use practices; (ii) assess awareness of antimicrobial resistance and perceptions regarding the contribution of dairy farming to AMR; (iii) determine the level of familiarity with SDCT; (iv) identify barriers and facilitators influencing SDCT implementation; (v) evaluate perceptions and, where applicable, practical experience regarding the effects of SDCT on udder health, milk production, and treatment costs; and (vi) assess farmers’ willingness to implement and veterinarians’ willingness to recommend SDCT in routine dairy practice.

## 2. Materials and Methods

### 2.1. Study Design and Target Population

A cross-sectional questionnaire-based study was conducted between April and June 2026 to evaluate the knowledge, attitudes, perceptions, and practices of Romanian dairy farmers and veterinarians regarding AMR and SDCT. The study also aimed to identify factors influencing the acceptance of SDCT and to explore perceived barriers and facilitators for its implementation in Romanian dairy herds.

The target population consisted of dairy farmers actively involved in the management of dairy cattle farms and veterinarians providing clinical services to dairy herds in Romania. Participation was voluntary and restricted to respondents aged ≥18 years. Incomplete questionnaires and responses from individuals not involved in the dairy sector were excluded from the analysis. A total of 132 valid questionnaires were included, comprising 71 veterinarians and 61 dairy farmers.

### 2.2. Questionnaire Development and Distribution

The questionnaire was developed by the first author following an extensive review of the available literature regarding antimicrobial use, antimicrobial resistance, antimicrobial stewardship, and SDCT in dairy cattle. Particular attention was given to previously published questionnaires investigating farmers’ and veterinarians’ perceptions of AMU and AMR in Europe. Before distribution, the questionnaire was independently reviewed by two researchers with expertise in dairy cattle health and reproduction to assess its content, clarity, and relevance. Based on their feedback, minor revisions were made to improve the wording and overall clarity of the questionnaire. No formal pilot testing or psychometric validation was performed. The questionnaire was administered in Romanian. An English translation of the questionnaire is provided as Supplementary Material S1 to facilitate interpretation by international readers.

**Figure 1:**
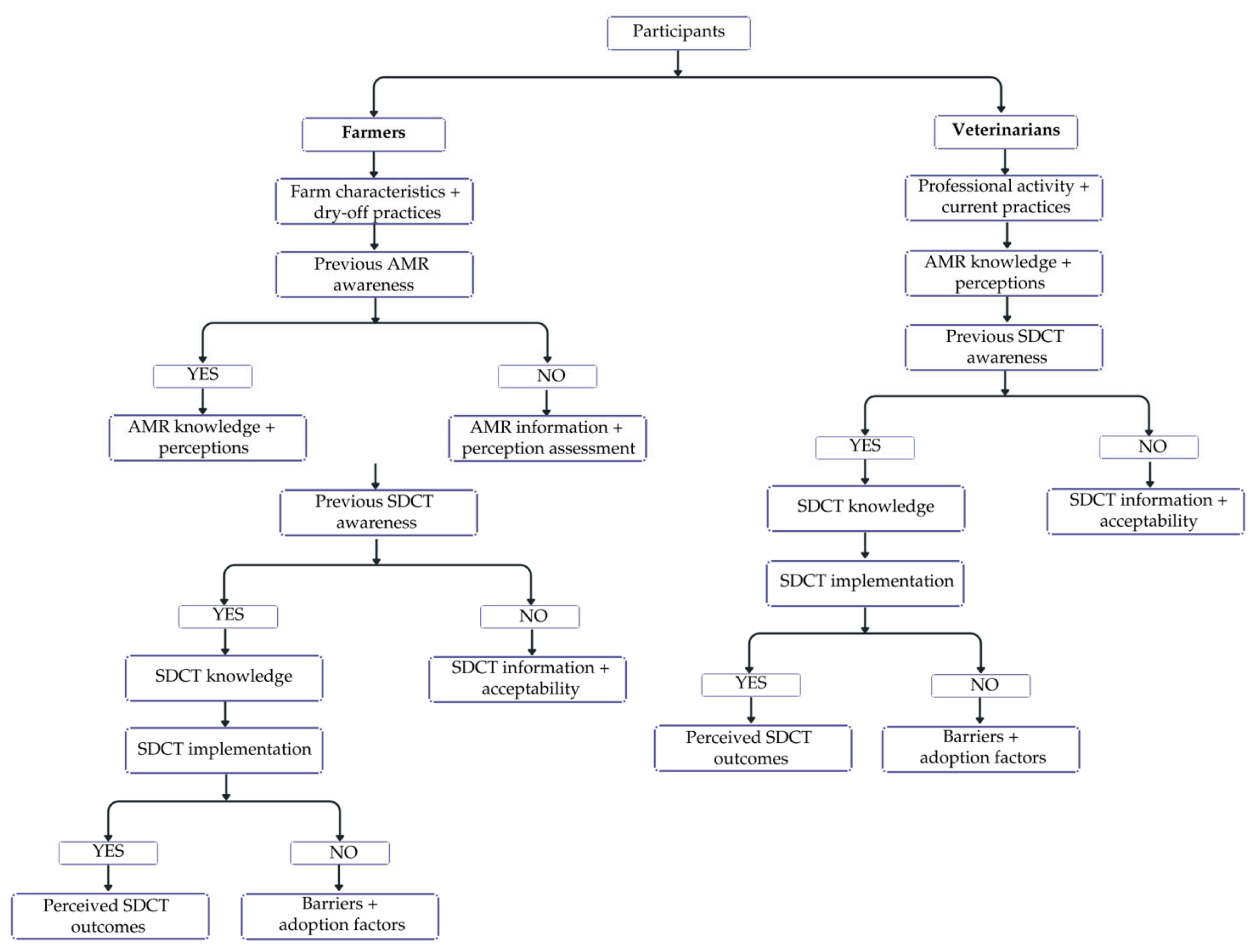
Structure and branching logic of the questionnaire used for dairy farmers and veterinarians. Participants were directed to different questionnaire sections according to their professional role and previous awareness of antimicrobial resistance (AMR) and selective dry cow therapy (SDCT). Respondents with previous SDCT experience completed additional questions regarding perceived implementation outcomes, whereas those without previous experience answered questions on barriers and facilitators for SDCT adoption. Participants unfamiliar with AMR or SDCT received standardized explanatory information before completing questions on perception and acceptance.

The questionnaire was created using Google Forms (Google LLC, Mountain View, CA, USA) and included a combination of single-choice questions, multiple-choice questions, Likert-scale statements, and semi-open questions. Branching logic was incorporated so that respondents were automatically directed to different sections depending on their previous answers, allowing the questionnaire to be adapted separately for dairy farmers and veterinarians (Figure 1). The farmer questionnaire consisted of 36 questions, whereas the veterinarian questionnaire included 39 questions.

The questionnaires covered the following thematic areas: demographic and professional characteristics; farm characteristics or veterinary practice characteristics; current dry-off management and antimicrobial use practices; knowledge and perceptions regarding antimicrobial resistance; previous awareness and understanding of SDCT; previous practical experience with SDCT and perceived outcomes; perceived barriers and facilitators for SDCT implementation; willingness to adopt or recommend SDCT in the future.

For respondents who indicated that they were unfamiliar with AMR or SDCT, the questionnaire provided a short standardized explanatory text describing the concept, objectives, and principles of antimicrobial resistance or selective dry cow therapy. The information was intentionally presented using neutral language and without persuasive statements to minimize response bias. After reading the information, participants continued with the remaining questions evaluating their perceptions and acceptance of SDCT.

Respondents who reported prior familiarity with SDCT completed the perception and acceptance questions based on their existing knowledge and experience. In contrast, respondents who were previously unfamiliar with SDCT first received the standardized explanatory information before answering the corresponding perception and acceptance questions. Therefore, these responses were obtained under different information contexts and should be interpreted accordingly rather than considered directly comparable.

A convenience sampling approach was used. The questionnaire was distributed electronically through professional dairy farming and veterinary networks using Facebook groups, WhatsApp groups, and e-mail between April and June 2026. Because the survey was disseminated through multiple online platforms and professional networks, the total number of individuals who received or viewed the invitation could not be determined; consequently, a response rate could not be calculated. Respondents originated from different regions across Romania. Participation was entirely voluntary and anonymous. No financial incentives or compensation were provided. Google Forms settings were configured to allow only one response per participant, thereby minimizing duplicate submissions. Completion of the questionnaire was considered to constitute informed consent for participation in the study.

### 2.3. Data Collection

Information collected from dairy farmers included demographic characteristics, farming experience, herd size, current dry-off practices, antimicrobial use during the dry period, use of bacteriological culture and SCC records, awareness of antimicrobial resistance, familiarity with SDCT, previous experience with SDCT, perceived barriers and facilitators for implementation, and willingness to adopt SDCT. Veterinarians were additionally asked about their professional experience, type of veterinary activity, recommendations regarding DCT, previous implementation of SDCT, perceived outcomes following SDCT adoption, and factors influencing their willingness to recommend SDCT.

### 2.4. SDCT Acceptance Score

To quantify respondents’ overall acceptance of SDCT, an SDCT acceptance score was calculated as the mean of four Likert-scale items evaluating: (i) confidence in SDCT as a dry-off strategy, (ii) perceived safety of SDCT for udder health, (iii) perceived contribution of SDCT to reducing antimicrobial use, and (iv) perceived feasibility of implementing SDCT in Romanian dairy farms. Each item was rated on a five-point Likert scale ranging from 1 (strongly disagree) to 5 (strongly agree). The SDCT acceptance score was calculated only for respondents who completed all four items and ranged from 1 to 5, with higher scores indicating greater acceptance of SDCT. The internal consistency of the four-item scale was assessed separately for farmers and veterinarians using Cronbach’s alpha.

### 2.5. Statistical Analysis

All statistical analyses were performed using R software version 4.5.3 (R Foundation for Statistical Computing, Vienna, Austria). Categorical variables were summarized as absolute frequencies and percentages, whereas continuous variables were presented as mean ± standard deviation (SD) or median and interquartile range (IQR), depending on data distribution. Associations between categorical variables were evaluated using the Chi-square test or Fisher’s exact test, when appropriate. Comparisons of continuous variables between two independent groups were performed using the Mann–Whitney U test, whereas comparisons involving more than two groups were conducted using the Kruskal–Wallis test. Factors associated with SDCT acceptance were evaluated in two stages. All candidate variables were initially evaluated using univariable analyses performed separately for farmers and veterinarians. Variables with *p* < 0.10 were subsequently entered into separate multivariable logistic regression models for each study group. For these analyses, the SDCT acceptance score was dichotomized into high SDCT acceptance (score ≥ 4) and lower SDCT acceptance (score < 4). To reduce the risk of model overfitting, only variables meeting the predefined selection criterion were retained, resulting in final models including two predictors for each study group. Adjusted odds ratios (ORs) and 95% confidence intervals (95% CIs) were calculated for all predictors retained in the final models. Multicollinearity was assessed using variance inflation factors (VIFs).

For Likert-scale questions, positive responses were defined as scores of 4 or 5. Percentages were calculated using all valid responses as the denominator unless otherwise specified.

Statistical significance was established at *p* < 0.05.

### 2.6. Ethical Considerations

Participation in the study was voluntary and anonymous. No personally identifiable information was collected, and all responses were treated confidentially and used exclusively for research purposes. The study was conducted in accordance with the ethical principles governing research involving human participants and was approved by the appropriate institutional ethics committee (Approval No. 560/08.04.2026).

## 3. Results

### 3.1. Characteristics of Respondents

The survey included 132 respondents, comprising 61 dairy farmers (46.2%) and 71 veterinarians (53.8%). Among farmers, most respondents had more than 10 years of dairy farming experience (78.7%), nearly half managed herds with fewer than 20 lactating cows (47.5%), and regular collaboration with a veterinarian was common (88.5%). Among veterinarians, more than half had over 10 years of professional experience (54.9%), while 59.2% regularly provided veterinary services to more than 10 dairy farms (Table 1).

### 3.2. Current Dry-Off Treatment Practices and Decision-Making Processes

Among dairy farmers, decisions regarding dry-off treatment were most commonly made jointly by the farmer and veterinarian (37.7%), followed by the farmer alone (34.4%) and the veterinarian alone (27.9%). Regarding current dry-off practices, administration of antibiotics only to selected cows was the most frequently reported strategy (41.0%), whereas 39.3% of farmers reported not applying any dry-off treatment. BDCT was reported by 11.5% of respondents, while 8.2% indicated that they were not directly involved in treatment decisions.

Among veterinarians, nearly half of the respondents (47.9%) reported being directly involved in treatment decisions at dry-off, whereas 22.5% collaborated with farmers and 22.5% primarily provided treatment recommendations. Regarding the treatment strategies recommended or implemented in practice, administration of antibiotics only to selected cows was also the most frequently reported approach (47.9%), followed by no dry-off treatment (25.4%) and BDCT (19.7%). The remaining 7.0% indicated that they were not directly involved in dry-off treatment decisions (Table 2). Although administration of antibiotics only to selected cows was the most frequently reported dry-off strategy in both respondent groups, this finding should be interpreted with caution. The questionnaire did not assess whether cow selection was based on evidence-based SDCT criteria, such as individual somatic cell count, clinical mastitis history, bacteriological culture results, or standardized treatment protocols. Therefore, these responses may also reflect empirical or experience-based selective treatment decisions rather than formal implementation of evidence-based SDCT.

### 3.3. Awareness and Perceptions Regarding Antimicrobial Resistance

Among dairy farmers, 43 of 61 respondents (70.5%) reported previous awareness of AMR, whereas 18 (29.5%) were unfamiliar with the concept before completing the questionnaire (Table 3). Among farmers who were aware of AMR, self-rated knowledge was generally favorable, with 46.5% rating their knowledge as good and 7.0% as very good, whereas only 4.7% considered their knowledge to be limited.

To ensure a common understanding of the concept, farmers who were initially unfamiliar with AMR received a brief neutral explanation before completing the subsequent perception-related questions.

**Figure 2:**
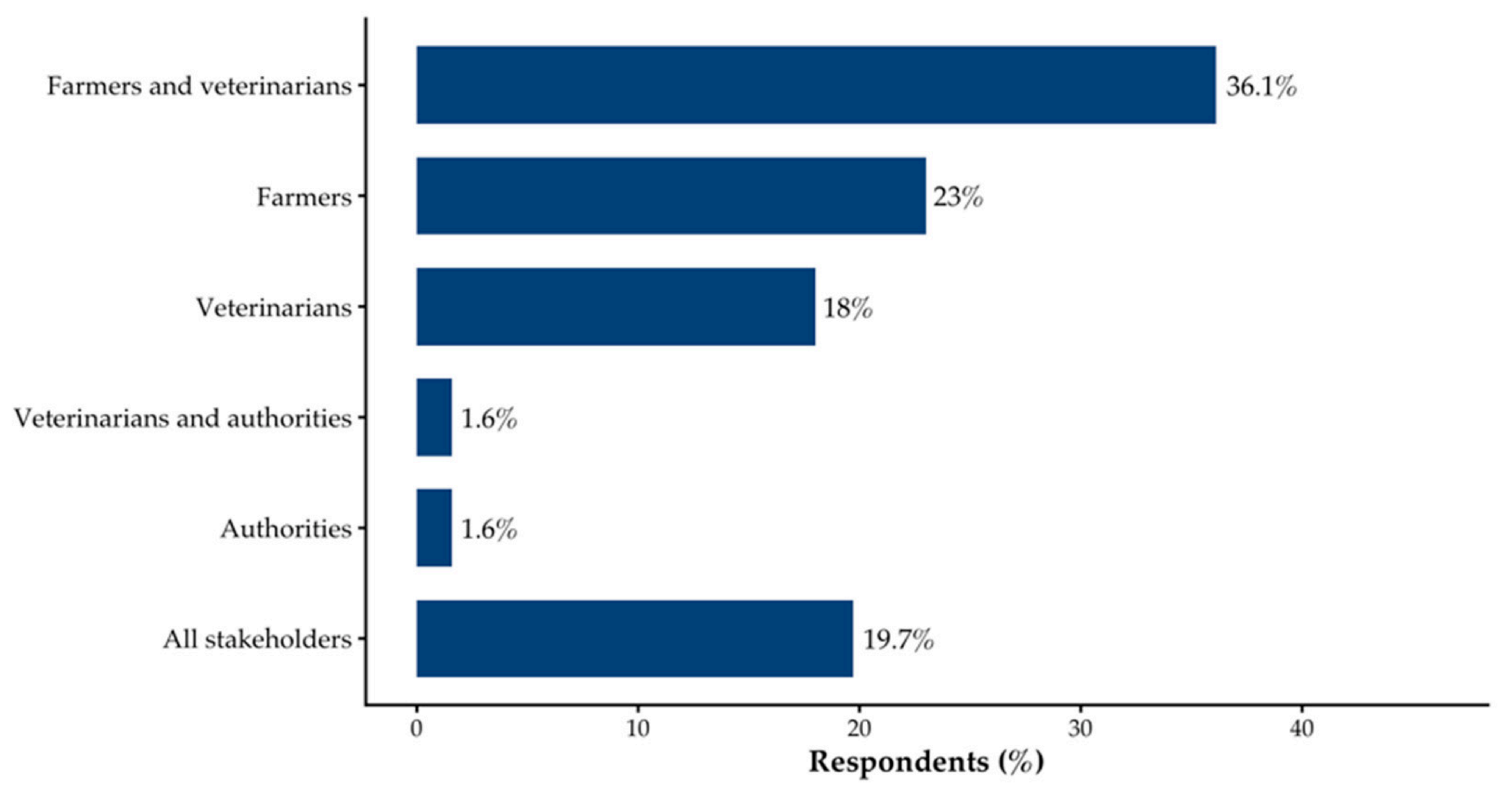
Perceived responsibility for reducing antimicrobial use among dairy farmers (*n* = 61). Responses indicate the stakeholder(s) considered primarily responsible for reducing antimicrobial use in dairy production.

Across all farmers (*n* = 61), concern regarding AMR was generally high, with 52.4% reporting high or very high levels of concern (Likert scores 4–5). Likewise, 60.7% considered that AMU in dairy farms substantially contributes to the development of AMR, and 77.0% agreed that reducing AMU in dairy farming is necessary. Regarding responsibility for reducing AMU, the largest proportion of farmers (36.1%) considered this to be a shared responsibility between farmers and veterinarians, followed by farmers alone (23.0%) and all stakeholders (19.7%) (Figure 2).

**Figure 3:**
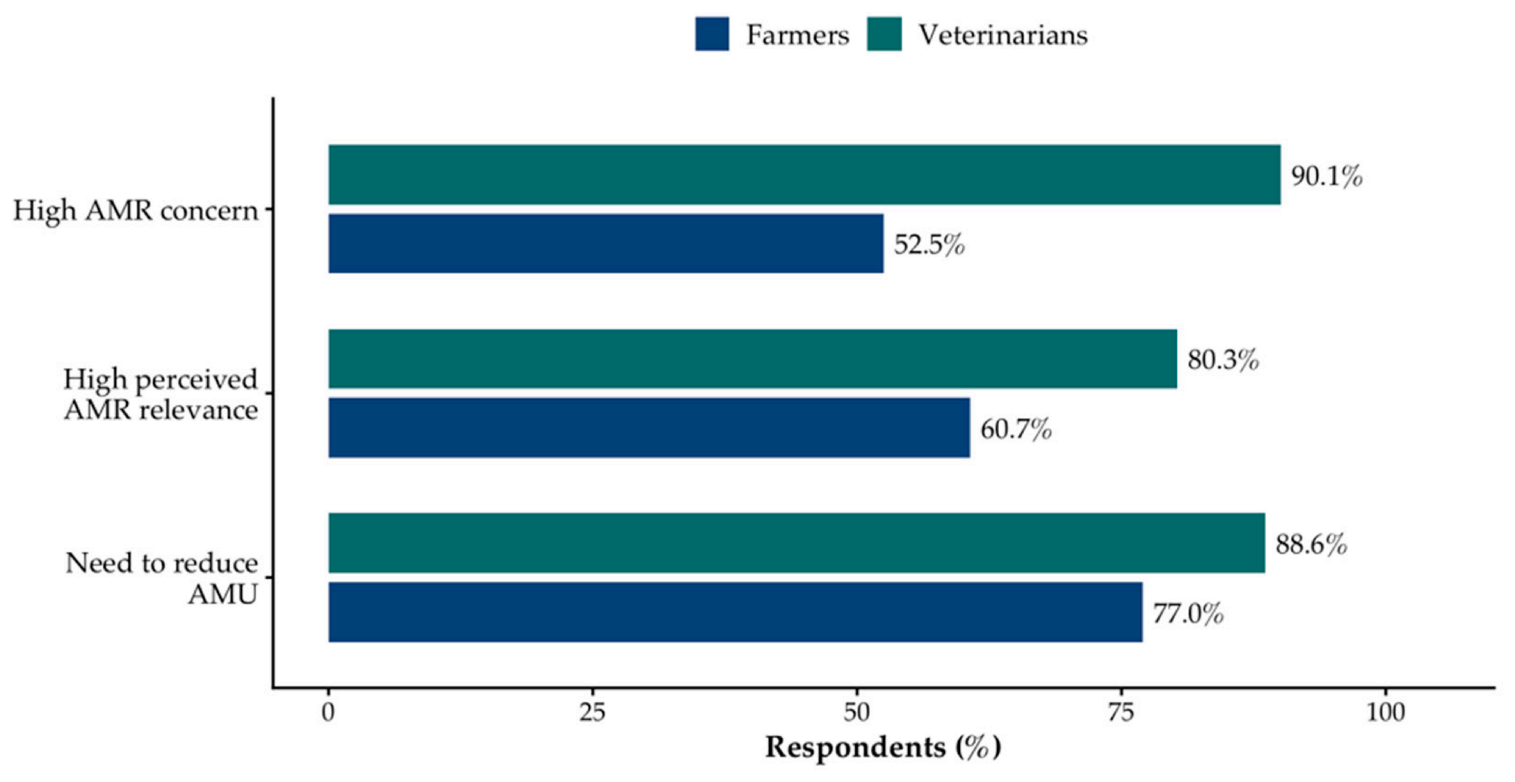
Comparison of key perceptions regarding AMR between dairy farmers (*n* = 61) and veterinarians (*n* = 71). High AMR concern and high perceived relevance of AMR in dairy farming correspond to Likert scores of 4 or 5 on a five-point scale. The “Need to reduce AMU” indicator represents respondents selecting “Agree” or “Strongly agree” (scores 4–5). Percentages were calculated using all valid responses as the denominator.

A direct comparison of AMR-related perceptions between dairy farmers and veterinarians is presented in Figure 3. Veterinarians reported higher levels of concern regarding AMR than farmers (90.1% vs. 52.5% reporting Likert scores of 4–5). Likewise, 80.3% of veterinarians considered AMR to be a major issue in dairy farming, whereas 60.7% of farmers believed that antimicrobial use in dairy farms substantially contributes to AMR development. Agreement that antimicrobial use should be reduced in dairy production was also higher among veterinarians (88.6%) than among farmers (77.0%).

### 3.4. Awareness and Perceptions Regarding SDCT

A total of 27.9% (17/61) of dairy farmers and 42.3% (30/71) of veterinarians reported previous awareness of SDCT (Table 4). Among respondents familiar with SDCT, self-rated knowledge was predominantly good or moderate in both groups. Among veterinarians, moderate knowledge was the most frequently reported level (63.3%), followed by good knowledge (26.7%). Previous practical experience with SDCT was reported by 70.6% of farmers familiar with the concept and by 83.3% of veterinarians who had previously heard of SDCT (Table 4). Among farmers already familiar with SDCT, veterinarians represented the most frequently reported source of information, whereas veterinarians primarily reported acquiring knowledge through professional courses or conferences and discussions with colleagues.

**Figure 4:**
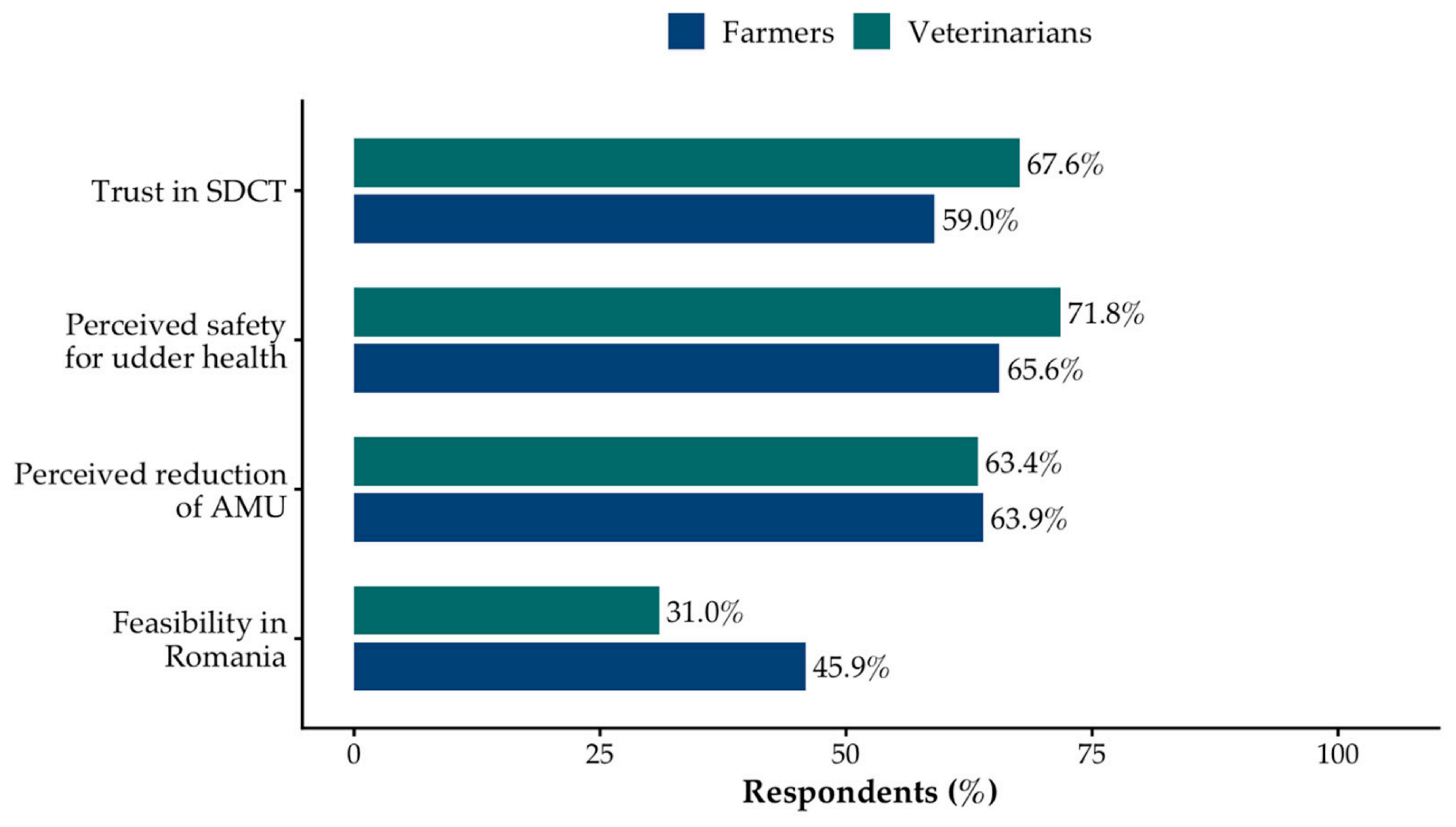
Comparison of positive perceptions regarding selective dry cow therapy (SDCT) between dairy farmers (*n* = 61) and veterinarians (*n* = 71). Bars represent the percentage of respondents selecting Likert scores of 4 or 5 (“Agree” or “Strongly agree”) for each statement. Percentages were calculated using all valid responses as the denominator.

Figure 4 summarizes respondents’ perceptions regarding SDCT after accounting for both previously informed participants and those who completed the informational section of the questionnaire. Overall, perceptions were generally favorable in both groups. Veterinarians reported slightly higher levels of trust in SDCT (67.6% vs. 59.0%) and were more likely to consider the strategy safe for udder health (71.8% vs. 65.6%). Both groups similarly recognized the potential of SDCT to reduce antimicrobial use (63.4–63.9% positive responses). In contrast, farmers expressed greater confidence than veterinarians regarding the feasibility of implementing SDCT under Romanian dairy farming conditions (45.9% vs. 31.0%).

After reading the standardized information on SDCT, 59.1% of farmers reported that they would be willing or very willing to adopt the strategy, while an additional 34.1% expressed moderate willingness. Likewise, 68.3% of veterinarians indicated that they would be willing or very willing to recommend SDCT following the informational section of the questionnaire. Overall, both respondent groups expressed favorable perceptions of SDCT and a high willingness to adopt or recommend the strategy following the provision of standardized information.

### 3.5. Practical Experience with SDCT and Perceived Outcome

Among respondents with previous practical experience of SDCT, both farmers and veterinarians reported their perceived outcomes following implementation or recommendation of the strategy. Among the 12 farmers who had previously implemented SDCT, 9 (75.0%) described their overall experience as positive or very positive, whereas 22 of the 25 veterinarians (88.0%) reported positive practical outcomes (Table 5).

Internal teat sealants were routinely incorporated into SDCT protocols by approximately two-thirds of respondents in both groups (66.7% of farmers and 72.0% of veterinarians). Perceived udder health outcomes were generally favorable. Half of the farmers (50.0%) and more than half of the veterinarians (56.0%) reported a perceived decrease in mastitis incidence following SDCT implementation, whereas the remaining respondents generally perceived no substantial change rather than an increase in mastitis occurrence.

Perceptions regarding production performance were also positive. Most farmers reported that milk yield was perceived to remain stable or increase after adopting SDCT, whereas veterinarians more frequently indicated that they were unable to reliably assess changes in milk production.

Perceived economic and antimicrobial stewardship outcomes were likewise favorable. Reduced treatment costs were reported by 58.3% of farmers and 44.0% of veterinarians, while respondents in both groups generally agreed that SDCT contributed to reduced antimicrobial use. Importantly, all farmers with previous SDCT experience expressed willingness to continue using the strategy, and 76.0% of veterinarians indicated that they would further expand its implementation in clinical practice. Overall, respondents with previous SDCT experience reported predominantly positive clinical, economic, and practical outcomes (Table 5).

### 3.6. Barriers and Facilitators to SDCT Implementation

Among the five farmers who had previously heard of SDCT but had not implemented the strategy, the main reasons for non-adoption were insufficient knowledge (80.0%) and the lack of herd-level diagnostic information, including individual somatic cell count records and bacteriological testing (40.0%). Similar perceptions were observed among farmers who were initially unfamiliar with SDCT but received standardized information during the questionnaire (*n* = 44), in whom insufficient information (34.1%), concerns regarding increased mastitis risk (22.7%), and the lack of veterinary recommendation (20.5%) were the most frequently perceived barriers. Regarding factors that could encourage future implementation, farmers consistently emphasized the need for convincing evidence that SDCT does not increase mastitis incidence or reduce milk yield, together with recommendations from their herd veterinarian and successful experiences from other farms. Despite their limited previous experience, most respondents expressed a positive attitude towards future SDCT adoption.

Among the five veterinarians who had not previously recommended SDCT, the principal barriers were the lack of herd-level data required for cow selection (60.0%) and farmer resistance (60.0%), followed by concerns regarding mastitis risk and lack of time (40.0%). Similarly, veterinarians who were initially unfamiliar with SDCT (*n* = 41) most frequently identified insufficient farmer training (29.3%), additional implementation costs (24.4%), lack of standardized protocols (14.6%), and farmer resistance (14.6%) as the main obstacles. Across both veterinarian groups, the availability of clear standardized implementation protocols, stronger scientific evidence supporting SDCT, improved access to bacteriological diagnostics, and greater farmer acceptance were considered the most important measures to facilitate wider implementation. Overall, respondents identified limited knowledge, insufficient herd-level diagnostic information, and the absence of practical implementation protocols as the principal barriers to SDCT adoption, while veterinary guidance and evidence demonstrating that SDCT can reduce AMU without compromising udder health were considered the strongest facilitators.

### 3.7. Factors Associated with SDCT Acceptance

To evaluate factors associated with acceptance of SDCT, an SDCT acceptance score was calculated as the mean of four perception domains (trust in SDCT, perceived safety, perceived reduction in AMU, and perceived feasibility of implementation). Internal consistency of the score was high among farmers (Cronbach’s α = 0.89) and acceptable among veterinarians (Cronbach’s α = 0.76), supporting the reliability of the composite measure. Overall, the median SDCT acceptance score did not differ between farmers and veterinarians (median = 3.75 for both groups; Wilcoxon rank-sum test, *p* = 0.633), indicating a comparable overall level of acceptance.

Among farmers, respondents who reported previous awareness of AMR demonstrated significantly higher SDCT acceptance scores than those without AMR awareness (median 4.00 vs. 3.38; Wilcoxon rank-sum test, *p* = 0.045). In contrast, previous awareness of the SDCT concept itself was not associated with higher acceptance (median 4.00 vs. 3.50; *p* = 0.250).

Acceptance of SDCT increased progressively with increasing concern regarding AMR (Kruskal–Wallis test, *p* < 0.001). Farmers expressing the highest level of concern exhibited the highest acceptance scores (median = 4.50), whereas respondents reporting little concern showed substantially lower acceptance (median = 2.75). Likewise, farmers who considered reducing AMU necessary reported significantly higher SDCT acceptance than those who considered reduction unnecessary or were uncertain (Kruskal–Wallis test, *p* = 0.012). Differences were also observed according to perceptions of responsibility for reducing antimicrobial use (Kruskal–Wallis test, *p* = 0.026), although no consistent trend across response categories was evident.

Among veterinarians, higher SDCT acceptance scores were associated with perceiving AMR as an important problem (Kruskal–Wallis test, *p* = 0.018) and with greater concern regarding AMR (Kruskal–Wallis test, *p* = 0.026). No significant associations were identified between SDCT acceptance and the perceived need to reduce antimicrobial use or any demographic or professional characteristic. The results of all univariable analyses are summarized in Table 6.

**Figure 5:**
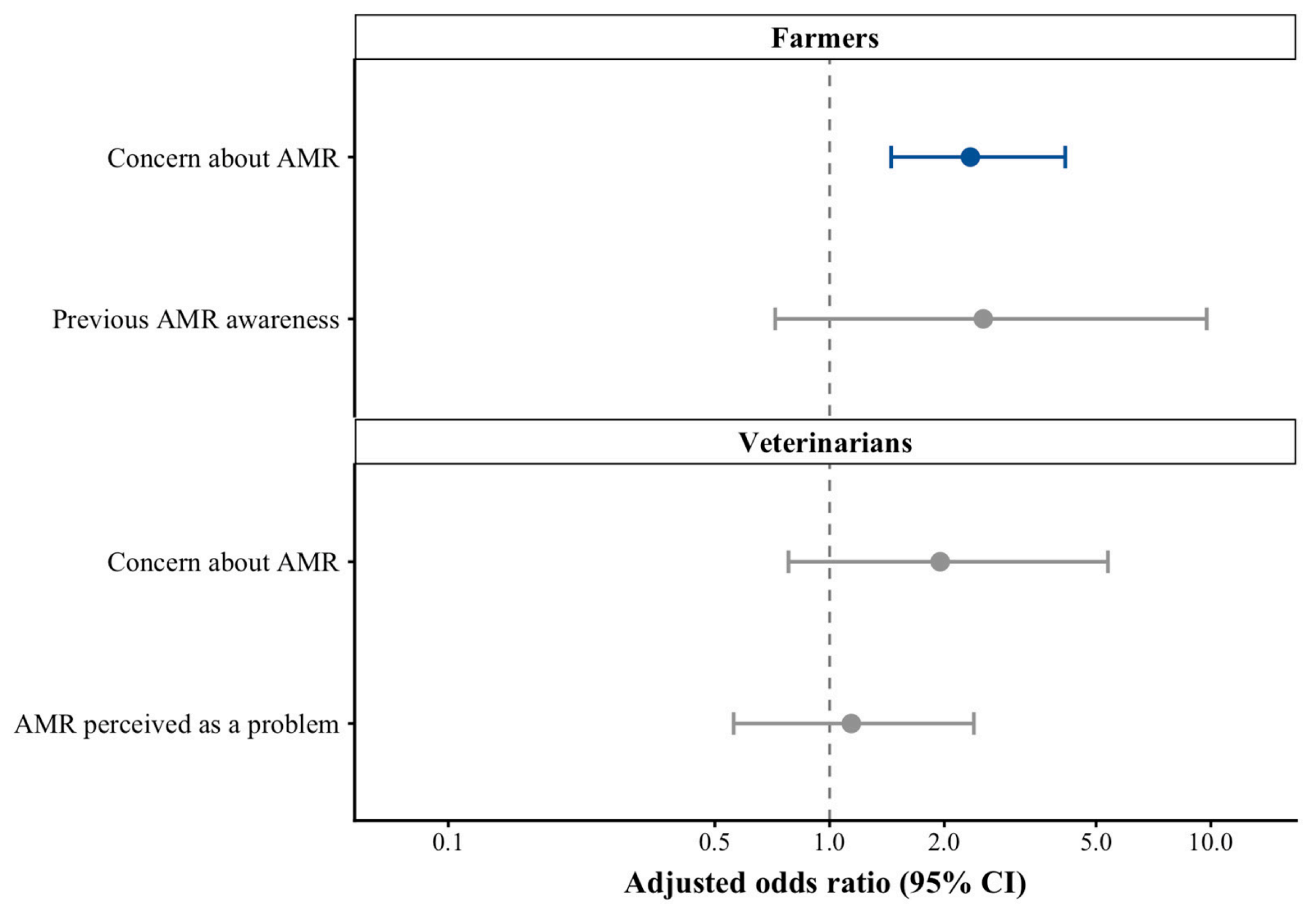
Multivariable logistic regression analysis of factors associated with high acceptance of SDCT among farmers and veterinarians. Forest plot showing adjusted odds ratios (ORs) and 95% confidence intervals (95% CIs) for factors associated with high SDCT acceptance in separate multivariable logistic regression models fitted for farmers and veterinarians. The dashed vertical line indicates an odds ratio of 1 (no association). Odds ratios greater than 1 indicate increased odds of high SDCT acceptance, whereas odds ratios below 1 indicate reduced odds. Among farmers, greater concern about AMR was the only independent predictor of high SDCT acceptance (OR = 2.34, 95% CI: 1.45–4.15, *p* = 0.001), whereas previous AMR awareness was not independently associated with high SDCT acceptance (OR = 2.53, 95% CI: 0.72–9.75, *p* = 0.157). Among veterinarians, neither concern about AMR nor perceiving AMR as an important problem remained independently associated with high SDCT acceptance. Blue symbols indicate statistically significant associations (*p* < 0.05), whereas gray symbols indicate non-significant associations.

To identify independent predictors of high SDCT acceptance, separate multivariable logistic regression models were fitted for farmers and veterinarians (Table 7; Figure 5). Among farmers, concern regarding AMR remained the only independent predictor of high SDCT acceptance. Each one-point increase in AMR concern was associated with more than a twofold increase in the odds of high SDCT acceptance (OR = 2.34, 95% CI: 1.45–4.15, *p* = 0.001), whereas previous awareness of AMR was not independently associated with high SDCT acceptance after adjustment (OR = 2.53, 95% CI: 0.72–9.75, *p* = 0.157). Among veterinarians, neither perceiving AMR as an important problem (OR = 1.14, 95% CI: 0.56–2.39, *p* = 0.719) nor concern regarding AMR (OR = 1.95, 95% CI: 0.78–5.37, *p* = 0.169) remained independently associated with high SDCT acceptance.

## 4. Discussion

To our knowledge, this is the first study to simultaneously evaluate the knowledge, attitudes, perceptions, and acceptance of SDCT among both dairy farmers and veterinarians in Romania. Overall, the findings indicate that although awareness of AMR was relatively high in both stakeholder groups, familiarity with SDCT remained limited, particularly among farmers. Nevertheless, after receiving standardized information describing the principles and objectives of SDCT, most respondents expressed positive attitudes toward the strategy and reported willingness to adopt or recommend its implementation. Importantly, although several AMR-related variables were associated with SDCT acceptance in the univariable analyses, only concern regarding AMR remained an independent predictor after multivariable adjustment. In contrast, previous awareness of AMR was no longer independently associated with high SDCT acceptance. These findings suggest that behavioural intention to adopt SDCT is driven less by simple awareness of AMR and more by the perceived importance of antimicrobial stewardship. These findings emphasize the importance of integrating antimicrobial stewardship messages into future implementation strategies while reinforcing the central role of veterinarians in promoting SDCT as one component of broader herd health management and prudent antimicrobial use at dry-off.

Among the surveyed dairy farmers, awareness of AMR was generally encouraging, with more than two-thirds of respondents reporting previous familiarity with the concept and most considering the reduction in AMU necessary. Veterinarians demonstrated even greater awareness, with almost all respondents expressing high concern regarding AMR and supporting antimicrobial stewardship. These findings are broadly consistent with previous studies from the United Kingdom and Switzerland, where dairy farmers and veterinarians increasingly recognize AMR as an important challenge for livestock production, although knowledge levels remain heterogeneous. Higham et al. [32] reported that dairy farmers maintaining closer contact with veterinarians demonstrated greater awareness of AMR and more responsible AMU practices, highlighting the educational role of veterinary practitioners. Similarly, Borelli et al. [33] found that Scottish dairy farmers generally acknowledged the importance of AMR, although important differences remained according to educational background and information sources. Comparable observations were reported by Schwendner et al. [34] among Swiss dairy farmers, who showed that although awareness of AMR was widespread, misconceptions persisted regarding antimicrobial susceptibility testing, critically important antimicrobials, and the mechanisms underlying AMR.

Compared with studies conducted in low- and middle-income countries, Romanian respondents demonstrated substantially higher awareness of AMR. Kallu et al. [35] reported that many Ethiopian dairy producers continued to purchase antibiotics without veterinary prescription, interrupted treatments prematurely, or failed to observe withdrawal periods despite having heard about antimicrobial resistance. In contrast, the relatively high awareness observed in the present study likely reflects the increasing dissemination of antimicrobial stewardship principles throughout Europe together with the implementation of European regulations aimed at reducing antimicrobial consumption in food-producing animals.

Despite this encouraging awareness of AMR, previous knowledge of SDCT remained remarkably limited. Fewer than one-third of farmers and less than half of veterinarians had previously heard about SDCT before completing the questionnaire. These findings suggest that although antimicrobial stewardship has become a familiar concept within Romanian dairy farming, its practical application through evidence-based strategies such as SDCT has not yet been sufficiently disseminated. Similar observations have been reported during the early implementation of SDCT in other European countries. Orpin [9] reported that although British dairy farmers generally understood the importance of reducing AMU, many remained unfamiliar with the practical aspects of SDCT or lacked confidence in applying it. Likewise, Higham et al. [32] observed that while SDCT was increasingly promoted within antimicrobial stewardship initiatives, many farmers still relied primarily on veterinarians for guidance regarding dry-off decisions. Collectively, these findings indicate that increasing awareness of AMR does not necessarily translate into familiarity with specific antimicrobial reduction strategies.

Interestingly, administration of antibiotics only to selected cows was the most frequently reported dry-off strategy among both farmers and veterinarians. A considerable proportion of farmers reported using this selective approach despite not being familiar with the SDCT concept itself, suggesting that selective treatment decisions may already be applied empirically rather than according to standardized SDCT protocols. This finding indicates that some Romanian farmers may have intuitively adopted elements of selective therapy based on individual clinical judgement or veterinary advice, even in the absence of formal SDCT guidelines. However, without standardized cow-selection criteria based on SCC records, mastitis history, or bacteriological testing, these practices cannot be considered true evidence-based SDCT. This distinction highlights an important opportunity for future educational programmes, as many farmers may already be receptive to SDCT principles but require structured protocols to ensure consistent and appropriate implementation.

Another noteworthy finding was the relatively high proportion of respondents reporting no dry-off treatment. Because the questionnaire did not investigate the reasons underlying this response, several explanations are possible. This finding may reflect the predominance of small or low-input dairy farms among the surveyed farmers, differences in management practices, limited access to dry cow therapy, or varying interpretations of the questionnaire. In addition, some respondents may have interpreted “no dry-off treatment” as the absence of intramammary antibiotic therapy while still applying non-antimicrobial measures, such as internal teat sealants or other management practices. Consequently, these responses should be interpreted with caution, and further studies are needed to better characterize dry-off management practices in Romanian dairy herds.

An important finding of the present study is that limited previous knowledge of SDCT did not prevent respondents from developing favourable perceptions once standardized information was provided. After reading a brief evidence-based description of SDCT, most farmers expressed willingness to adopt the strategy, while an even greater proportion of veterinarians indicated willingness to recommend it in practice. This observation suggests that resistance to SDCT implementation may be driven more by limited exposure to reliable information than by fundamental opposition to reducing AMU. Similar conclusions have been proposed by Lam et al. [29], who argued that behavioural change among dairy farmers depends not only on technical knowledge but also on effective communication strategies tailored to farmers’ individual needs and concerns. Rather than assuming that farmers reject innovation, veterinary advisors should focus on providing clear, practical, and herd-specific information capable of increasing confidence in SDCT implementation.

Interestingly, previous awareness of SDCT itself was not independently associated with greater acceptance of the strategy. Instead, farmers expressing greater concern regarding AMR demonstrated significantly higher SDCT acceptance scores, and AMR concern remained the only independent predictor after multivariable adjustment. This finding provides important insight into the mechanisms underlying behavioural change. It suggests that farmers are primarily motivated by understanding the broader implications of AMU for animal and public health rather than by simple familiarity with a particular management strategy. Similar observations have been reported by Borelli et al. [33], who found that attitudes toward AMR, rather than factual knowledge alone, were more closely associated with prudent AMU behaviours. Likewise, Higham et al. [32] demonstrated that farmers recognizing the importance of antimicrobial stewardship were more receptive to interventions aimed at reducing antimicrobial consumption. Collectively, these findings indicate that future educational programmes should emphasize not only how SDCT is implemented but also why reducing AMU is essential for preserving antimicrobial efficacy and maintaining sustainable dairy production.

The identification of barriers to SDCT implementation provides valuable insight into why this strategy has not yet been widely adopted in Romanian dairy herds. Among respondents who had no previous experience with SDCT, the most frequently perceived obstacle was insufficient information regarding the strategy, followed by concerns about increased mastitis risk, lack of veterinary recommendation, and the absence of standardized implementation protocols. Veterinarians additionally emphasized insufficient farmer training and limited access to herd-level diagnostic information. Similar challenges have been consistently reported across Europe, suggesting that the barriers associated with SDCT implementation are remarkably similar despite differences in dairy production systems. Fear of increased mastitis remains one of the most frequently reported concerns associated with SDCT. Orpin [9] reported that many British dairy farmers feared higher mastitis incidence, increased SCC, or even cow losses if antibiotics were omitted at dry-off, despite the growing body of scientific evidence supporting selective treatment in appropriately selected low-risk cows. Similarly, Higham et al. [32] and Borelli et al. [33] reported that limited knowledge, lack of confidence, and reliance on veterinary guidance represented important obstacles to adopting antimicrobial reduction strategies. Schwendner et al. [34] further showed that successful implementation of prudent AMU was associated with greater use of diagnostic testing and herd-specific treatment protocols. In the present study, however, insufficient information represented an even greater barrier than fear of mastitis, suggesting that Romanian farmers are not fundamentally opposed to SDCT but rather require greater confidence and practical guidance before implementing the strategy under routine farm conditions.

Respondents also highlighted several facilitators that could substantially increase SDCT adoption. Farmers most frequently requested convincing scientific evidence demonstrating that SDCT does not increase mastitis incidence or reduce milk yield, together with recommendations from their herd veterinarian. Veterinarians, in turn, emphasized the importance of standardized implementation protocols, locally generated scientific evidence, rapid bacteriological diagnostics, and improved farmer education. These findings are consistent with previous European studies, which similarly identified veterinary guidance, scientific evidence, and practical implementation support as key facilitators of SDCT adoption [9,32].

Successful SDCT implementation relies on reliable herd-level diagnostic information, including individual SCC, bacteriological culture, and mastitis history. Similar findings have been reported previously, highlighting that access to diagnostic data is essential for evidence-based cow selection and prudent antimicrobial use [34]. These observations emphasize the importance of improving diagnostic infrastructure alongside educational initiatives.

An encouraging observation was that respondents with previous practical experience of SDCT generally reported favourable perceived outcomes. Most farmers and veterinarians described positive overall experiences, widespread use of ITS, stable or improved milk production, reduced treatment costs, and either unchanged or reduced mastitis incidence. Furthermore, nearly all respondents with previous SDCT experience indicated that they intended to continue implementing or recommending the strategy. These findings are consistent with numerous controlled clinical studies demonstrating that appropriately selected low-risk cows can be dried off without antimicrobials without compromising udder health [13,14,15,16,17,18,19,20,21,22]. They also complement the observations of Orpin [9], who reported that farmers initially expressed uncertainty regarding SDCT but became increasingly confident after observing satisfactory results within their own herds. Collectively, these findings suggest that practical experience substantially reduces the perceived risks associated with SDCT and strengthens long-term acceptance. However, these findings should be interpreted cautiously because they reflect respondents’ perceptions and experiences rather than objectively measured herd-level outcomes. The questionnaire did not distinguish whether these perceptions were based on herd records, personal clinical observations, or feedback received from farmers.

The prominent role attributed to veterinarians throughout the present study further reinforces previous evidence that veterinary practitioners are central to successful antimicrobial stewardship programmes. Farmers familiar with SDCT most frequently identified veterinarians as their primary source of information, while veterinarians themselves reported obtaining knowledge mainly through continuing professional education and communication with colleagues. These findings agree with international studies demonstrating that veterinarians remain the most trusted advisors regarding antimicrobial use decisions. Higham et al. [32] found that regular veterinary involvement was consistently associated with more responsible AMU, whereas Borelli et al. [33,36] reported that farmers relying primarily on veterinarians for antimicrobial information demonstrated better knowledge and more prudent AMU practices. Similarly, Llanos-Soto et al. [27] showed that veterinarians recognized their pivotal role in antimicrobial stewardship while simultaneously acknowledging barriers such as farmer expectations, inconsistent communication, limited time, and insufficient diagnostic support.

Communication will undoubtedly play a central role in the successful implementation of SDCT. Lam et al. [11] emphasized that behavioural change depends not only on transferring technical knowledge but also on understanding farmers’ motivations, concerns, and readiness to adopt new management practices. Accordingly, tailored communication, herd-specific recommendations, regular follow-up, and continuous feedback are likely to be more effective than generic educational campaigns. Consistent messaging across veterinarians, universities, advisory services, dairy organizations, and regulatory authorities may further strengthen farmers’ confidence in SDCT implementation.

The present findings have several practical implications for antimicrobial stewardship in Romania. Increasing awareness through veterinary-led education, improving access to SCC monitoring and bacteriological diagnostics, and developing standardized national SDCT protocols could substantially facilitate wider implementation of the strategy. Collectively, these measures could support the transition from blanket antimicrobial use toward evidence-based SDCT as part of a broader antimicrobial stewardship strategy that also includes effective mastitis prevention, herd health management, and evidence-based antimicrobial use, without compromising udder health or farm profitability.

This study has several strengths. To our knowledge, it is the first survey evaluating knowledge, perceptions, and acceptance of SDCT among both dairy farmers and veterinarians in Romania. Including both stakeholder groups provided complementary perspectives on the factors influencing SDCT implementation and allowed direct comparison between those responsible for herd management and those providing veterinary guidance. Furthermore, the use of multivariable regression analysis enabled the identification of independent predictors of SDCT acceptance rather than relying solely on descriptive comparisons.

Several limitations should be acknowledged. First, the cross-sectional design precludes establishing causal relationships between perceptions and SDCT acceptance. Second, the study employed a voluntary convenience sampling approach, and because the questionnaire was distributed through multiple online platforms and professional networks, the response rate could not be calculated. Consequently, respondents with a particular interest in antimicrobial stewardship or SDCT may have been overrepresented. In addition, all information regarding dry-off practices, previous SDCT experience, and perceived implementation outcomes was self-reported and was not verified using objective farm records or clinical data, making the results susceptible to recall and social desirability bias. Furthermore, respondents who were previously unfamiliar with SDCT received standardized explanatory information before completing the perception and acceptance questions. Although this information was intentionally presented in neutral language, it may have influenced their immediate responses. Consequently, willingness to adopt or recommend SDCT among these respondents should be interpreted as an initial response to standardized information rather than as a definitive predictor of future implementation. The questionnaire also did not investigate the criteria used to select cows for antimicrobial treatment, such as somatic cell count, clinical mastitis history, bacteriological culture, or standardized SDCT protocols. Therefore, it was not possible to determine whether the reported selective antimicrobial use represented evidence-based SDCT or empirical selective treatment. Finally, although respondents originated from different regions across Romania, the predominance of smaller dairy herds may limit the generalizability of these findings to larger commercial dairy operations. Future nationally representative surveys and longitudinal studies are needed to confirm these observations.

## 5. Conclusions

This study provides the first assessment of the knowledge, perceptions, and acceptance of SDCT among a sample of Romanian dairy farmers and veterinarians. Although awareness of AMR was generally high, many farmers already reported administering antimicrobials only to selected cows at dry-off. However, familiarity with the formal concept and terminology of evidence-based SDCT remained limited, particularly among farmers, indicating that selective antibiotic treatment was often applied empirically rather than according to standardized SDCT criteria. Nevertheless, after receiving standardized information, most respondents expressed positive attitudes toward SDCT and willingness to adopt or recommend the strategy, suggesting that limited knowledge rather than resistance to change is the main barrier to its wider implementation. Furthermore, concern regarding AMR was the only independent predictor of SDCT acceptance among farmers, emphasizing the importance of antimicrobial stewardship awareness in promoting behavioural change. Overall, these findings suggest that successful implementation of SDCT in Romanian dairy farming could be supported by veterinary-led education, improved access to herd-level diagnostics, standardized national protocols, and effective farmer–veterinarian communication. Together, these measures could support wider adoption of evidence-based dry cow therapy while reducing unnecessary antimicrobial use and promoting sustainable dairy production. Because this study was based on a voluntary convenience sample, these findings should be interpreted as reflecting the perceptions of the surveyed respondents rather than all Romanian dairy farmers and veterinarians.

## Figures and Tables

**Table 1:** Characteristics of survey respondents.

Variable	Category	*n* (%)
Respondent type	Farmers	61 (46.2)
	Veterinarians	71 (53.8)
Farmers (*n* = 61)		
Herd size	<20 cows	29 (47.5)
	20–50 cows	14 (23.0)
	51–100 cows	14 (23.0)
	>100 cows	4 (6.6)
Experience	<5 years	6 (9.8)
	5–10 years	7 (11.5)
	>10 years	48 (78.7)
Veterinary collaboration	Regular	54 (88.5)
	Occasional	6 (9.8)
	None	1 (1.6)
Veterinarians (*n* = 71)		
Professional experience	<5 years	17 (23.9)
	5–10 years	15 (21.1)
	>10 years	39 (54.9)
Farms regularly served	1–5	23 (32.4)
	6–10	6 (8.5)
	>10	42 (59.2)
Work with dairy cattle	Yes	50 (70.4)
	Occasionally	16 (22.5)
	No	5 (7.0)

Data are presented as number of respondents (*n*) and percentage (%). Percentages may not total exactly 100% because of rounding.

**Table 2:** Current dry-off treatment practices and decision-making among survey respondents.

Variable	Category	*n* (%)
Farmers (*n* = 61)		
Decision-maker for dry-off treatment	Farmer only	21 (34.4)
	Veterinarian only	17 (27.9)
	Farmer and veterinarian jointly	23 (37.7)
Current dry-off treatment strategy	Antibiotics administered only to selected cows	25 (41.0)
	No dry-off treatment	24 (39.3)
	Blanket dry cow therapy (BDCT)	7 (11.5)
	Unknown/not involved	5 (8.2)
Veterinarians (*n* = 71)		
Role in dry-off treatment decisions	Decide directly	34 (47.9)
	Collaborate with farmer	16 (22.5)
	Recommend treatment	16 (22.5)
	Not directly involved	5 (7.0)
Recommended dry-off treatment strategy	Antibiotics administered only to selected cows	34 (47.9)
	No dry-off treatment	18 (25.4)
	Blanket dry cow therapy (BDCT)	14 (19.7)
	Not involved in decision-making	5 (7.0)

Data are presented as number of respondents (*n*) and percentage (%). Percentages may not total exactly 100% because of rounding.

**Table 3:** Awareness of antimicrobial resistance (AMR) and self-rated knowledge among dairy farmers.

Variable	Category	*n* (%)
Heard of antimicrobial resistance (AMR)	Yes	43 (70.5)
	No	18 (29.5)
Self-rated knowledge of AMR *	Limited	2 (4.7)
	Moderate	18 (41.9)
	Good	20 (46.5)
	Very good	3 (7.0)

Awareness of AMR among dairy farmers (*n* = 61). * Self-rated knowledge of AMR was assessed only among respondents who had previously heard of AMR (*n* = 43).

**Table 4:** Awareness, self-rated knowledge, and previous experience with SDCT.

Variable	Category	Farmers, *n* (%)	Veterinarians, *n* (%)
Previously aware of SDCT	Yes	17 (27.9)	30 (42.3)
	No	44 (72.1)	41 (57.7)
Self-rated knowledge *	Good	8 (47.1)	8 (26.7)
	Moderate	8 (47.1)	19 (63.3)
	Limited	1 (5.9)	2 (6.7)
	Very limited	–	1 (3.3)
Previous practical experience with SDCT **	Yes	12 (70.6)	25 (83.3)
	No	5 (29.4)	5 (16.7)

* Percentages calculated only among respondents who had previously heard of SDCT. ** Percentages calculated only among respondents familiar with SDCT.

**Table 5:** Reported outcomes following previous implementation of SDCT.

Outcome	Farmers (*n* = 12)	Veterinarians (*n* = 25)
Positive overall experience	9/12 (75.0%)	22/25 (88.0%)
Internal teat sealant used	8/12 (66.7%)	18/25 (72.0%)
Reported perceived decrease in mastitis	6/12 (50.0%)	14/25 (56.0%)
Reported perceived unchanged or increased milk yield	9/12 (75.0%)	13/25 (52.0%)
Reported perceived reduction in treatment costs	7/12 (58.3%)	11/25 (44.0%)
Would continue/scale-up SDCT	12/12 (100%)	19/25 (76.0%)

Percentages were calculated only among respondents with previous practical SDCT experience.

**Table 6:** Univariable analyses of factors associated with SDCT acceptance among dairy farmers and veterinarians.

Respondent Group	Variable	Statistical Test	*p*-Value
Farmers	AMR awareness	Mann–Whitney U	0.045
	SDCT awareness	Mann–Whitney U	0.250
	AMR concern	Kruskal–Wallis	<0.001
	Need to reduce antimicrobial use	Kruskal–Wallis	0.012
	Responsibility for AMR	Kruskal–Wallis	0.026
	Farming experience	Kruskal–Wallis	0.600
	Herd size	Kruskal–Wallis	0.695
	Veterinary collaboration	Kruskal–Wallis	0.663
	Self-rated AMR knowledge	Kruskal–Wallis	0.162
	Previous SDCT implementation	Mann–Whitney U	0.423
Veterinarians	AMR as a problem	Kruskal–Wallis	0.018
	AMR concern	Kruskal–Wallis	0.026
	Need to reduce antimicrobial use	Kruskal–Wallis	0.509
	Professional experience	Kruskal–Wallis	>0.05
	Work with dairy cattle	Kruskal–Wallis	>0.05
	Number of farms served	Kruskal–Wallis	>0.05
	Previous SDCT recommendation	Mann–Whitney U	>0.05

Results of univariable analyses evaluating factors associated with SDCT acceptance. Variables with *p* < 0.10 were considered for inclusion in the multivariable logistic regression models.

**Table 7:** Multivariable logistic regression models evaluating factors associated with high acceptance of SDCT.

Respondent Group	Predictor	Adjusted OR (95% CI)	*p*-Value
Farmers	Previous AMR awareness (Yes vs. No)	2.53 (0.72–9.75)	0.157
	AMR concern (per 1-point increase)	2.34 (1.45–4.15)	0.001
Veterinarians	AMR perceived as a problem (per 1-point increase)	1.14 (0.56–2.39)	0.719
	AMR concern (per 1-point increase)	1.95 (0.78–5.37)	0.169

Results of separate multivariable logistic regression models evaluating predictors of high SDCT acceptance among dairy farmers and veterinarians. Odds ratios (ORs) are adjusted for the variables retained in each final model. Statistical significance was defined as *p* < 0.05.

## Data Availability

The English version of the questionnaire is available as Supplementary Material S1. The anonymized survey dataset supporting the findings of this study is available as Supplementary Material S2.

## Supplementary Materials

The following supporting information can be downloaded at: https://www.mdpi.com/article/10.3390/ani16152401/s1. Supplementary Material S1. English version of the questionnaire used in the survey; Supplementary Material S2. Anonymized survey dataset used for the statistical analyses.

## References

[1] Ut I.D., Berean D.I., Bogdan L.M., Ciupe S., Gog Bogdan S. (2025). Selective Dry Cow Therapy in Modern Dairy Management: Balancing Udder Health and Antimicrobial Stewardship. Vet. Sci..

[2] Chantziaras I., Boyen F., Callens B., Dewulf J. (2014). Correlation between Veterinary Antimicrobial Use and Antimicrobial Resistance in Food-Producing Animals: A Report on Seven Countries. J. Antimicrob. Chemother..

[3] Freimuth V., Linnan H.W., Potter P. (2000). Communicating the Threat of Emerging Infections to the Public. Emerg. Infect. Dis..

[4] Kuipers A., Koops W.J., Wemmenhove H. (2016). Antibiotic Use in Dairy Herds in the Netherlands from 2005 to 2012. J. Dairy Sci..

[5] Biggs A. (2017). Update on Dry Cow Therapy 1. Antibiotic v Non-Antibiotic Approaches. In Pract..

[6] Higgins H.M., Golding S.E., Mouncey J., Nanjiani I., Cook A.J.C. (2017). Understanding Veterinarians’ Prescribing Decisions on Antibiotic Dry Cow Therapy. J. Dairy Sci..

[7] Brunton L.A., Duncan D., Coldham N.G., Snow L.C., Jones J.R. (2012). A Survey of Antimicrobial Usage on Dairy Farms and Waste Milk Feeding Practices in England and Wales. Vet. Rec..

[8] Contiero B., Cozzi G., Lora I., Gottardo F. (2025). Transition to Selective Dry Cow Therapy for Responsible Antimicrobial Use in Dairy Cattle: A Case Study. Animal.

[9] Orpin P. (2017). What Can We Learn from Farmers Experiences and Attitudes to Selective Dry Cow Therapy?. Cattle Pract..

[10] Erskine R.J., Eberhart R.J., Hutchinson L.J., Spencer S.B., Campbell M.A. (1988). Incidence and Types of Clinical Mastitis in Dairy Herds with High and Low Somatic Cell Counts. J. Am. Vet. Med. Assoc..

[11] Lam T.J.G.M., Jansen J., Wessels R.J. (2017). The RESET Mindset Model Applied on Decreasing Antibiotic Usage in Dairy Cattle in the Netherlands. Ir. Vet. J..

[12] Riekerink R.G.M.O., Barkema H.W., Kelton D.F., Scholl D.T. (2008). Incidence Rate of Clinical Mastitis on Canadian Dairy Farms. J. Dairy Sci..

[13] Rowe S.M., Godden S.M., Nydam D.V., Gorden P.J., Lago A., Vasquez A.K., Royster E., Timmerman J., Thomas M.J. (2020). Randomized Controlled Trial Investigating the Effect of 2 Selective Dry-Cow Therapy Protocols on Udder Health and Performance in the Subsequent Lactation. J. Dairy Sci..

[14] Rowe S.M., Godden S.M., Nydam D.V., Gorden P.J., Lago A., Vasquez A.K., Royster E., Timmerman J., Thomas M.J. (2020). Randomized Controlled Non-Inferiority Trial Investigating the Effect of 2 Selective Dry-Cow Therapy Protocols on Antibiotic Use at Dry-off and Dry Period Intramammary Infection Dynamics. J. Dairy Sci..

[15] Lipkens Z., Piepers S., De Vliegher S. (2023). Impact of Selective Dry Cow Therapy on Antimicrobial Consumption, Udder Health, Milk Yield, and Culling Hazard in Commercial Dairy Herds. Antibiotics.

[16] Vasquez A.K., Nydam D.V., Foditsch C., Wieland M., Lynch R., Eicker S., Virkler P.D. (2018). Use of a Culture-Independent on-Farm Algorithm to Guide the Use of Selective Dry-Cow Antibiotic Therapy. J. Dairy Sci..

[17] Filippone Pavesi L., Pollera C., Sala G., Cremonesi P., Monistero V., Biscarini F., Bronzo V. (2023). Effect of the Selective Dry Cow Therapy on Udder Health and Milk Microbiota. Antibiotics.

[18] McParland S., Dillon P.G., Flynn J., Ryan N., Arkins S., Kennedy A. (2019). Effect of Using Internal Teat Sealant with or without Antibiotic Therapy at Dry-off on Subsequent Somatic Cell Count and Milk Production. J. Dairy Sci..

[19] Cameron M., Keefe G.P., Roy J.P., Stryhn H., Dohoo I.R., McKenna S.L. (2015). Evaluation of Selective Dry Cow Treatment Following On-Farm Culture: Milk Yield and Somatic Cell Count in the Subsequent Lactation. J. Dairy Sci..

[20] Cameron M., McKenna S.L., MacDonald K.A., Dohoo I.R., Roy J.P., Keefe G.P. (2014). Evaluation of Selective Dry Cow Treatment Following On-Farm Culture: Risk of Postcalving Intramammary Infection and Clinical Mastitis in the Subsequent Lactation. J. Dairy Sci..

[21] Vanhoudt A., van Hees-Huijps K., van Knegsel A.T.M., Sampimon O.C., Vernooij J.C.M., Nielen M., van Werven T. (2018). Effects of Reduced Intramammary Antimicrobial Use during the Dry Period on Udder Health in Dutch Dairy Herds. J. Dairy Sci..

[22] Ut I.D., Berean D.I., Bogdan L.M., Ciupe S., Gog-Bogdan S. (2026). Internal Teat Sealant as an Alternative to Intramammary Antibiotics at Dry-Off in Low-Risk Dairy Cows: Effects on Udder Health, Milk Yield, Antimicrobial Use, and Economic Outcomes. Animals.

[23] Kabera F., Dufour S., Keefe G., Cameron M., Roy J.P. (2020). Evaluation of Quarter-Based Selective Dry Cow Therapy Using Petrifilm on-Farm Milk Culture: A Randomized Controlled Trial. J. Dairy Sci..

[24] OsterA O., Salverad L. (2009). Norwegian mastitis control programme. Ir. Vet. J..

[25] Santman-Berends I.M.G.A., van den Heuvel K.W.H., Lam T.J.G.M., Scherpenzeel C.G.M., van Schaik G. (2021). Monitoring Udder Health on Routinely Collected Census Data: Evaluating the Short- to Mid-Term Consequences of Implementing Selective Dry Cow Treatment. J. Dairy Sci..

[26] Krattley-Roodenburg B., Huybens L.J., Nielen M., van Werven T. (2021). Dry Period Management and New High Somatic Cell Count during the Dry Period in Dutch Dairy Herds under Selective Dry Cow Therapy. J. Dairy Sci..

[27] Llanos-Soto S.G., Vezeau N., Wemette M., Bulut E., Greiner Safi A., Moroni P., Shapiro M.A., Ivanek R. (2021). Survey of Perceptions and Attitudes of an International Group of Veterinarians Regarding Antibiotic Use and Resistance on Dairy Cattle Farms. Prev. Vet. Med..

[28] Jones P.J., Marier E.A., Tranter R.B., Wu G., Watson E., Teale C.J. (2015). Factors Affecting Dairy Farmers’ Attitudes towards Antimicrobial Medicine Usage in Cattle in England and Wales. Prev. Vet. Med..

[29] Lam T.J.G.M., Jansen J., van den Borne B.H.P., Renes R.J., Hogeveen H. (2011). What Veterinarians Need to Know about Communication to Optimise Their Role as Advisors on Udder Health in Dairy Herds. N. Z. Vet. J..

[30] Scherpenzeel C.G.M., den Uijl I.E.M., van Schaik G., Riekerink R.G.M.O., Hogeveen H., Lam T.J.G.M. (2016). Effect of Different Scenarios for Selective Dry-Cow Therapy on Udder Health, Antimicrobial Usage, and Economics. J. Dairy Sci..

[31] Wemette M., Safi A.G., Beauvais W., Ceres K., Shapiro M., Moroni P., Welcome F.L., Ivanek R. (2020). New York State Dairy Farmers’ Perceptions of Antibiotic Use and Resistance: A Qualitative Interview Study. PLoS ONE.

[32] Higham L.E., Deakin A., Tivey E., Porteus V., Ridgway S., Rayner A.C. (2018). A Survey of Dairy Cow Farmers in the United Kingdom: Knowledge, Attitudes and Practices Surrounding Antimicrobial Use and Resistance. Vet. Rec..

[33] Borelli E., Ellis K., Pamphilis N.M., Tomlinson M., Hotchkiss E. (2023). Factors Influencing Scottish Dairy Farmers’ Antimicrobial Usage, Knowledge and Attitude towards Antimicrobial Resistance. Prev. Vet. Med..

[34] Schwendner A.A., Lam T.J.G.M., Bodmer M., Cousin M.E., Schüpbach-Regula G., van den Borne B.H.P. (2020). Knowledge, Attitude and Practices of Swiss Dairy Farmers towards Intramammary Antimicrobial Use and Antimicrobial Resistance: A Latent Class Analysis. Prev. Vet. Med..

[35] Kallu S.A., Kebede N., Kassa T., Wubaye A.M., Kainga H., Mekonnen H., Simuunza M.C. (2024). Knowledge, Attitudes, Practices, and Risk Perception of Antimicrobial Use and Antimicrobial Resistance Among Dairy Farm Owners/Workers in Addis Ababa, Ethiopia. Infect. Drug Resist..

[36] Borelli E., Ellis K., Tomlinson M., Hotchkiss E. (2023). Antimicrobial Usage and Resistance in Scottish Dairy Herds: A Survey of Farmers’ Knowledge, Behaviours and Attitudes. BMC Vet. Res..

