# Supplementary material for: Knowledge, Perceptions and Acceptance of Selective Dry Cow Therapy Among Romanian Dairy Farmers and Veterinarians
Source: Animals (Basel). 2026 Aug 4;16(15):2401. doi: 10.3390/ani16152401 (PMC13464846; doi:10.3390/ani16152401)
Supplement: Supplementary file 1 [file animals-16-02401-s001.zip › Supplementary Material S1.pdf]

Supplementary Material S1

English translation of the questionnaire used in the study

The questionnaire was originally developed and administered in Romanian. The English version provided below is a translation prepared for publication and is included to facilitate interpretation by international readers.

Study title

Knowledge, perceptions and acceptance of selective dry cow therapy (SDCT) among Romanian dairy farmers and veterinarians.

This questionnaire is part of a doctoral research project investigating strategies to reduce antimicrobial use in dairy cattle through the implementation of selective dry cow therapy (SDCT). The purpose of this study is to evaluate the knowledge, perceptions, and willingness of dairy farmers and veterinarians to adopt this approach. Participation in this study is entirely voluntary. Your responses are anonymous, and no personally identifiable information will be collected. The information provided will be used exclusively for scientific research purposes and may be published in scientific journals or presented at scientific meetings only in an anonymized and aggregated form. You may discontinue the questionnaire at any time before submission without any consequences. By completing and submitting this questionnaire, you confirm that you have read the above information and voluntarily agree to participate in this study.

Question 1

Respondent category

Please select one answer.

○ **Farmer** *(continue to Question 2)*

○ **Veterinarian** *(continue to Question 65)*

Questionnaire for Dairy Farmers

Question 2

In which county is your farm located? *

Question 3

How many lactating cows do you currently have on your farm? *

Please select one answer.

○ Fewer than 20

○ 20–50

○ 51–100

○ More than 100

Question 4

How many years have you been involved in dairy farming? *

Please select one answer.

○ Less than 5 years

○ 5–10 years

○ More than 10 years

Question 5

Do you regularly collaborate with a veterinarian?

Please select one answer.

○ Yes

○ No

○ Occasionally

Question 6

How would you assess the level of mastitis problems on your farm? *

Please select one answer.

○ It is not a problem.

○ It is a problem, but it is under control.

○ It becomes a problem during certain periods.

○ It is a major problem.

○ I do not know / I cannot assess.

Question 7

Do you consider that the occurrence of mastitis is influenced by cow management at dry-off?

Please select one answer.

○ No

○ Yes, to a small extent

○ Yes, to a great extent

○ I do not know

Question 8

How concerned are you about the occurrence of mastitis during the dry period or after calving?

Please select one answer.

○ Not at all

○ Slightly

○ Moderately

○ Very

○ Extremely

Question 9

What type of treatment do you routinely use at dry-off? *

Please select one answer.

○ Antibiotic treatment for all cows

○ No treatment is administered

○ Antibiotic treatment only for selected cows

○ I do not know / I am not involved in the decision

○ Other (please specify): ______________________

Question 10

Who decides the dry-off treatment used on your farm? *

Please select one answer.

○ Myself

○ The veterinarian

○ Jointly (farmer and veterinarian)

○ Other (please specify): ______________________

Question 11

How comfortable would you feel NOT administering antibiotics to all cows at dry-off?

Please select one answer.

○ Not comfortable at all

○ Slightly comfortable

○ Moderately comfortable

○ Comfortable

○ Very comfortable

Farmers' Knowledge and Perceptions of Antimicrobial Resistance

Question 12

Have you heard of antimicrobial resistance (AMR)?

Please select one answer.

○ Yes *(continue to Question 13)*

○ No *(continue to Question 20)*

Question 13

How would you rate your level of knowledge regarding antimicrobial resistance?

Please select one answer.

○ Very poor

○ Poor

○ Moderate

○ Good

○ Very good

Question 14

To what extent are you concerned about the spread of antimicrobial resistance?

Please select one answer.

1 = Not at all

2

3

4

5 = To a very great extent

Question 15

To what extent do you believe that the use of antibiotics on dairy farms contributes to the development of antimicrobial resistance?

Please select one answer.

1 = Not at all

2

3

4

5 = Very much

Farmers' Knowledge and Perceptions of Antimicrobial Resistance (continued)

Question 16

Do you consider that reducing antibiotic use on dairy farms is necessary? *

Please select one answer.

○ Yes

○ No

○ I do not know

Question 17

Who do you believe has the primary responsibility for reducing antibiotic use in the dairy sector?

(You may select more than one answer.)

☐ Farmers

☐ Veterinarians

☐ Authorities

☐ All of the above

☐ I do not know

Question 18

Have you attempted to reduce antibiotic use on your farm?

Please select one answer.

○ Yes

○ No *(continue to Question 26)*

○ I have not tried yet, but I am interested *(continue to Question 26)*

Question 19

If YES, what measures have you implemented to reduce antibiotic use?

(Select all that apply.)

☐ Improved farm hygiene

☐ Monitoring udder health

☐ More frequent consultation with the veterinarian

☐ Reduction of preventive antibiotic treatments

☐ Implementation of Selective Dry Cow Therapy (SDCT)

☐ Bacteriological examination and antimicrobial susceptibility testing to guide targeted antimicrobial therapy

☐ Other (please specify): ______________________

Information on Antimicrobial Resistance and Related Perceptions

Antimicrobial resistance (AMR) is the ability of bacteria to become resistant to antibiotics, making treatments less effective. Frequent or inappropriate use of antibiotics may contribute to the development of this phenomenon, with consequences for animal health and, indirectly, public health.

Question 20

After reading the explanation above, how clear is the concept of antimicrobial resistance to you? *

Please select one answer.

○ Not clear at all

○ Slightly clear

○ Moderately clear

○ Clear

○ Very clear

Question 21

To what extent are you concerned about the spread of antimicrobial resistance? *

Please select one answer.

1 = Not at all

2

3

4

5 = To a very great extent

Question 22

To what extent do you believe that the use of antibiotics on dairy farms contributes to the development of antimicrobial resistance?

Please select one answer.

1 = Not at all

2

3

4

5 = Very much

Question 23

Do you consider that reducing antibiotic use on dairy farms is necessary? *

Please select one answer.

○ Yes

○ No

○ I do not know

Question 24

Who do you believe has the primary responsibility for reducing antibiotic use in the dairy sector?

(You may select more than one answer.)

☐ Farmers

☐ Veterinarians

☐ Authorities

☐ All of the above

☐ I do not know

Question 25

After reading the explanation above, would you consider reducing antibiotic use on your farm?

Please select one answer.

○ Yes

○ No

Concept of Selective Dry Cow Therapy (SDCT)

Question 26

Have you heard of Selective Dry Cow Therapy (SDCT)? *

Please select one answer.

○ Yes *(continue to Question 38)*

○ No *(continue to Question 27)*

Information about SDCT and Assessment of Acceptability

Selective Dry Cow Therapy (SDCT) involves administering antibiotics only to cows at increased risk of intramammary infection at dry-off. Cows considered healthy do not receive antibiotic treatment; instead, internal teat sealants are generally used. Studies have shown that, when properly implemented, this approach can reduce antibiotic use without significantly affecting udder health or milk production.

Question 27

After reading this description, how clear is the concept of SDCT to you? *

Please select one answer.

○ Not clear at all

○ Slightly clear

○ Moderately clear

○ Clear

○ Very clear

Question 28

Following this explanation, to what extent would you trust this therapeutic approach?

Please select one answer.

1 = Not at all

2

3

4

5 = To a very great extent

Question 29

To what extent do you consider SDCT to be safe for udder health?

Please select one answer.

1 = Not safe at all

2

3

4

5 = Very safe

Question 30

To what extent do you believe that SDCT can contribute to reducing antibiotic use on dairy farms?

Please select one answer.

1 = Not at all

2

3

4

5 = To a very great extent

Question 31

To what extent would you be willing to try SDCT? *

Please select one answer.

○ Not willing at all

○ Slightly willing

○ Moderately willing

○ Willing

○ Very willing

Question 32

What do you consider to be the main obstacle to implementing SDCT? *

Please select one answer.

○ Fear of an increased risk of mastitis

○ Lack of sufficient information

○ Lack of recommendation from the veterinarian

○ Lack of clear implementation protocols

○ Additional costs

○ Additional workload

○ Other: ______________________

Question 33

What would convince you to implement SDCT on your farm? *

(Select all that apply.)

☐ Clear evidence that SDCT does not increase the risk of mastitis

☐ Clear evidence that SDCT does not reduce milk production

☐ Recommendation from the veterinarian

☐ Clear implementation protocols

☐ Access to diagnostic testing (SCC, bacteriology)

☐ Successful examples from other farms

☐ Reduced costs

☐ Legal requirements/regulations

☐ If it significantly reduces antibiotic use

☐ Other: ______________________

Question 34

How do you think SDCT would affect farm costs? *

Please select one answer.

○ Costs would increase significantly

○ Costs would increase slightly

○ No impact on costs

○ Costs would decrease

○ I do not know

Question 35

To what extent do you think implementing SDCT could increase the risk of mastitis?

Please select one answer.

1 = Not at all

2

3

4

5 = To a very great extent

Question 36

To what extent do you think SDCT can be successfully implemented on Romanian dairy farms?

Please select one answer.

1 = Not at all

2

3

4

5 = To a very great extent

Question 37

Please provide any comments or concerns regarding the use of SDCT.

Farmers' Knowledge of SDCT

Question 38

Where did you first learn about SDCT? *(You may select more than one answer.)* *

☐ From a veterinarian

☐ Professional courses/training

☐ At university

☐ From the Internet

☐ From other farmers

☐ From pharmaceutical companies

☐ Other: ______________________

Question 39

How would you rate your level of knowledge regarding SDCT? *

Please select one answer.

○ Very poor

○ Poor

○ Moderate

○ Good

○ Very good

Question 40

To what extent do you trust this therapeutic approach?

3

Please select one answer.

1 = Not at all

2

3

4

5 = To a very great extent

Question 41

To what extent do you consider SDCT to be safe for udder health? *

Please select one answer.

1 = Not safe at all

2

3

4

5 = Very safe

Question 42

To what extent do you believe that SDCT can contribute to reducing antibiotic use on dairy farms?

Please select one answer.

1 = Not at all

2

3

4

5 = To a very great extent

Question 43

To what extent do you believe that SDCT can be successfully implemented on dairy farms in Romania?

Please select one answer.

1 = Not at all

2

3

4

5 = To a very great extent

(Continue to Question 44.)

SDCT Implementation

Question 44

Have you ever implemented SDCT on your farm? *

Please select one answer.

○ Yes *(continue to Question 45)*

○ No *(continue to Question 57)*

Farmers' Experiences Following SDCT Implementation

Question 45

How would you evaluate the outcomes of SDCT implementation? *

Please select one answer.

○ Very negative

○ Negative

○ Neutral

○ Positive

○ Very positive

Question 46

For cows that did not receive antibiotic treatment at dry-off, did you use internal teat sealants?

Please select one answer.

○ Yes

○ No

○ I am not familiar with internal teat sealants

Question 47

How did SDCT affect the incidence of mastitis? *

Please select one answer.

○ Increased

○ No change

○ Decreased

○ I do not know

Question 48

How did SDCT affect milk production? *

Please select one answer.

○ Increased

○ No change

○ Decreased

○ I do not know

Question 49

To what extent did SDCT contribute to reducing antibiotic use on your farm?

Please select one answer.

1 = Not at all

2

3

4

5 = Very much

Question 50

How did implementing SDCT affect farm costs? *

Please select one answer.

○ Costs increased significantly

○ Costs increased slightly

○ No impact

○ Reduced costs

○ I do not know / Unable to assess

Question 51

What were the most challenging aspects of implementing SDCT on your farm? *(Select all that apply.)* *

☐ Identifying cows requiring treatment

☐ Lack of data (SCC, mastitis history)

☐ Limited access to diagnostic testing

☐ Correct implementation of the protocol

☐ Monitoring cows after dry-off

☐ Organizing farm activities

☐ There were no difficulties

☐ Other: ______________________

Question 52

What limitations or disadvantages do you consider SDCT to have? *(Select all that apply.)*

☐ Increased risk of mastitis

☐ Requires more monitoring

☐ Requires additional data (SCC, etc.)

☐ Requires more time and organization

☐ Requires veterinary support

☐ I do not consider SDCT to have any disadvantages

☐ Other: ______________________

Question 53

What benefits have you observed following SDCT implementation? *(Select all that apply.)*

☐ Reduced antibiotic use

☐ Lower treatment costs

☐ Similar udder health status

☐ More efficient treatment organization

☐ Greater confidence in treatment decisions

☐ I have not observed any benefits

☐ Other: ______________________

Question 54

Would you continue using SDCT in the future? *

Please select one answer.

○ Yes

○ No

○ It depends

Question 55

Would you recommend SDCT to other farmers? *

Please select one answer.

○ Yes

○ No

○ It depends

Question 56

Please provide any comments or concerns regarding the use of SDCT.

Barriers and Factors Influencing SDCT Adoption

Question 57

What are the main reasons why you have not implemented SDCT? *(Select all that apply.)*

☐ I am not sufficiently familiar with the concept

☐ I do not trust its effectiveness

☐ Fear of an increased risk of mastitis

☐ My veterinarian has not recommended it

☐ Lack of clear selection criteria

☐ Lack of diagnostic testing (SCC, bacteriological examinations, etc.)

☐ Other: ______________________

Question 58

What do you consider to be the main obstacle to implementing SDCT? *

Please select one answer.

○ Fear of an increased risk of mastitis

○ Lack of sufficient information

○ Lack of clear implementation protocols

○ Additional costs

○ Additional workload

○ Other: ______________________

Question 59

What would convince you to implement SDCT on your farm? *(Select all that apply.) **

☐ Clear evidence that SDCT does not increase the risk of mastitis

☐ Evidence that SDCT does not reduce milk production

☐ Recommendation from a veterinarian

☐ Clear implementation protocols

☐ Access to diagnostic testing (SCC, bacteriology)

☐ Successful examples from other farms

☐ Reduced dry-off costs

☐ Legal requirements/regulations

☐ If it significantly reduced antibiotic use

☐ Other: ______________________

Question 60

How do you think SDCT would affect farm costs? *

Please select one answer.

○ Costs would increase significantly

○ Costs would increase slightly

○ No impact

○ Costs would decrease

○ I do not know

Question 61

To what extent do you think implementing SDCT could increase the risk of mastitis? *

Please select one answer.

1 = Not at all

2

3

4

5 = To a very great extent

Question 62

To what extent do you think implementing SDCT could affect milk production? *

Please select one answer.

1 = Not at all

2

3

4

5 = To a very great extent

Question 63

To what extent would you be willing to try SDCT? *

Please select one answer.

○ Not willing at all

○ Slightly willing

○ Moderately willing

○ Willing

○ Very willing

Question 64

Please provide any comments or concerns regarding the use of SDCT.

Questionnaire for Veterinarians

Question 65

How many years have you been practicing veterinary medicine? *

Please select one answer.

○ Less than 5 years

○ 5–10 years

○ More than 10 years

Question 66

Do you currently work with dairy cattle? *

Please select one answer.

○ Yes

○ No

○ Occasionally

Question 67

How many dairy farms do you regularly provide veterinary services to?

Please select one answer.

○ 1–5

○ 6–10

○ More than 10

Question 68

To what extent are you involved in treatment decisions for dairy cattle?

Please select one answer.

○ I make the treatment decisions directly

○ I recommend treatments

○ I collaborate with the farmer

○ I am not directly involved

Question 69

Do you consider that the occurrence of mastitis is influenced by the management of cows at dry-off?

Please select one answer.

○ No

○ Yes, to a small extent

○ Yes, to a great extent

○ I do not know

Question 70

How concerned are you about the occurrence of mastitis during the dry period or after calving?

Please select one answer.

○ Not at all

○ Slightly

○ Moderately

○ Very

○ Extremely

Question 71

Which dry-off therapy do you routinely recommend or implement?

Please select one answer.

○ Antibiotic treatment for all cows

○ Antibiotic treatment only for selected cows

○ I do not recommend treatment

○ I am not involved in treatment decisions

○ Other: ______________________

Question 72

How comfortable would you feel NOT administering antibiotics to all cows at dry-off?

Please select one answer.

○ Not comfortable at all

○ Slightly comfortable

○ Moderately comfortable

○ Comfortable

○ Very comfortable

Question 73

To what extent do you consider antimicrobial resistance to be a problem in the dairy cattle sector?

Please select one answer.

1 = Not at all

2

3

4

5 = To a very great extent

Question 74

To what extent are you concerned about the spread of antimicrobial resistance? *

Please select one answer.

1 = Not at all

2

3

4

5 = To a very great extent

Question 75

Do you consider reducing antibiotic use in the dairy cattle sector to be necessary?

Please select one answer.

○ Yes

○ No

○ I do not know

Question 76

Have you implemented or recommended measures to reduce antibiotic use on dairy farms?

Please select one answer.

○ Yes

○ No

○ Partially

Question 77

If YES, what type of measures have you recommended? *(Select all that apply.)*

☐ Selective Dry Cow Therapy

☐ Improved hygiene

☐ SCC monitoring

☐ Bacteriological testing and antimicrobial susceptibility testing to guide targeted antimicrobial therapy

☐ Using antibiotics only for curative purposes

☐ Other: ______________________

Question 78

Have you heard of Selective Dry Cow Therapy (SDCT)? *

Please select one answer.

○ Yes *(continue to Question 79)*

○ No *(continue to Question 107)*

Veterinarians' Knowledge of SDCT

Question 79

Where did you first learn about SDCT? *(You may select more than one answer.)* *

☐ Scientific literature

☐ Courses/conferences

☐ University

☐ Colleagues/other veterinarians

☐ Pharmaceutical companies

☐ Other: ______________________

Question 80

How would you rate your level of knowledge regarding SDCT? *

Please select one answer.

○ Very poor

○ Poor

○ Moderate

○ Good

○ Very good

Question 81

To what extent do you trust this therapeutic approach? *

Please select one answer.

1 = Not at all

2

3

4

5 = To a very great extent

Question 82

To what extent do you consider SDCT to be safe for udder health? *

Please select one answer.

1 = Not safe at all

2

3

4

5 = Very safe

Question 83

To what extent do you believe that SDCT can contribute to reducing antibiotic use on dairy farms?

Please select one answer.

1 = Not at all

2

3

4

5 = To a very great extent

Question 84

To what extent do you consider farmers' reluctance to be a barrier to SDCT implementation?

Please select one answer.

1 = Not at all

2

3

4

5 = To a very great extent

Question 85

To what extent do you believe that SDCT can be successfully implemented on dairy farms in Romania?

Please select one answer.

1 = Not at all

2

3

4

5 = To a very great extent

SDCT Implementation

Question 86

Have you recommended or implemented SDCT on the farms you work with? *

Please select one answer.

○ Yes *(continue to Question 87)*

○ No *(continue to Question 99)*

○ Partially, where infection pressure is not a concern *(continue to Question 87)*

Outcomes of SDCT Implementation by Veterinarians

Question 87

On what type of farms have you implemented SDCT?

Please select one answer.

○ Small farms

○ Medium-sized farms

○ Large farms

○ On all types of farms

Question 88

How would you evaluate the outcomes of SDCT implementation? *

Please select one answer.

○ Very negative

○ Negative

○ Neutral

○ Positive

○ Very positive

Question 89

For cows that did not receive antibiotic treatment at dry-off, did you use or recommend the use of internal teat sealants?

Please select one answer.

○ Yes

○ No

○ I am not familiar with internal teat sealants

Question 90

How did SDCT affect the incidence of mastitis? *

Please select one answer.

○ Increased

○ No change

○ Decreased

○ I do not know

Question 91

How did SDCT affect milk production? *

Please select one answer.

○ Increased

○ No change

○ Decreased

○ I do not know

Question 92

To what extent did SDCT contribute to reducing antibiotic use on the farms where it was implemented?

Please select one answer.

1 = Not at all

2

3

4

5 = Very much

Question 93

How did SDCT implementation affect farm costs on the farms where it was implemented? *

Please select one answer.

○ Costs increased significantly

○ Costs increased slightly

○ No impact

○ Reduced costs

○ I do not know / Unable to assess

Question 94

What were the most challenging aspects of implementing SDCT on the farm? *(Select all that apply.) **

☐ Identifying cows requiring treatment

☐ Lack of data (SCC, mastitis history)

☐ Limited access to diagnostic testing

☐ Correct implementation of the protocol

☐ Collaboration with the veterinarian

☐ Monitoring cows after dry-off

☐ Organizing farm activities

☐ There were no difficulties

☐ Other: ______________________

Question 95

What limitations or disadvantages do you consider SDCT to have? *(Select all that apply.) **

☐ Increased risk of mastitis

☐ Requires more monitoring

☐ Requires additional data (SCC, etc.)

☐ Requires more time and organization

☐ Requires veterinary support

☐ I do not consider SDCT to have any disadvantages

☐ Other: ______________________

Question 96

What benefits have you observed following SDCT implementation? *(Select all that apply.) **

☐ Reduced antibiotic use

☐ Lower treatment costs

☐ Similar udder health status

☐ More efficient treatment organization

☐ Greater confidence in treatment decisions

☐ I have not observed any benefits

☐ Other: ______________________

Question 97

To what extent would you be willing to recommend wider implementation of SDCT on the farms you work with?

Please select one answer.

1 = Not at all

2

3

4

5 = To a very great extent

Question 98

Please provide any comments or concerns regarding the use of SDCT.

Barriers and Factors Influencing SDCT Adoption

Question 99

What are the main reasons why you have not recommended SDCT to farmers? *(Select all that apply.)*

☐ Lack of confidence in the effectiveness of the method

☐ Fear of an increased incidence of mastitis

☐ Lack of farm data (SCC, mastitis history, etc.)

☐ Limited access to diagnostic testing (bacteriological examination)

☐ Farmers' resistance

☐ I consider blanket antibiotic treatment at dry-off to be safer

☐ Lack of time for implementation

☐ Other: ______________________

Question 100

What do you consider to be the main obstacle to implementing SDCT? *

Please select one answer.

○ Lack of farm data

○ Insufficient farmer training

○ Fear of mastitis

○ Lack of standardized protocols

○ Additional workload

○ Potential additional costs

○ Farmers' opposition

○ Other: ______________________

Question 101

What would convince you to recommend or implement SDCT in the future? *(Select all that apply.)* *

☐ Scientific evidence demonstrating the successful implementation of SDCT on Romanian dairy farms

☐ Clear and standardized protocols

☐ Access to farm data (SCC, mastitis history)

☐ Access to rapid bacteriological diagnostics

☐ Farmer training

☐ Farmer acceptance

☐ Positive experiences from other farms

☐ Recommendations from official guidelines

☐ If required by law

☐ Other: ______________________

Question 102

How do you think SDCT would affect farm costs? *

Please select one answer.

○ Costs would increase significantly

○ Costs would increase slightly

○ No impact

○ Costs would decrease

○ I do not know / Unable to assess

Question 103

To what extent do you believe that implementing SDCT could increase the risk of mastitis? *

Please select one answer.

1 = Not at all

2

3

4

5 = To a very great extent

Question 104

To what extent do you believe that implementing SDCT could affect milk production? *

Please select one answer.

1 = Not at all

2

3

4

5 = To a very great extent

Question 105

To what extent would you be willing to recommend or implement SDCT? *

Please select one answer.

○ Not willing at all

○ Slightly willing

○ Moderately willing

○ Willing

○ Very willing

Information about SDCT and Acceptability Assessment

Selective Dry Cow Therapy (SDCT) involves administering antibiotics only to cows at increased risk of intramammary infections at dry-off, while healthy cows do not receive antibiotic treatment but are generally treated with internal teat sealants. Studies have shown that, when implemented correctly, this approach can reduce antibiotic use without significantly affecting udder health.

Question 106

After this description, how clear does the concept of SDCT seem to you? *

Please select one answer.

○ Not clear at all

○ Slightly clear

○ Moderately clear

○ Clear

○ Very clear

2

Question 107

After this explanation, to what extent would you trust this therapeutic approach? *

Please select one answer.

1 = Not at all

2

3

4

5 = To a very great extent

Question 108

To what extent do you consider SDCT to be safe for udder health? *

Please select one answer.

1 = Not safe at all

2

3

4

5 = Very safe

Question 109

To what extent do you consider that SDCT can contribute to reducing antibiotic use on dairy farms? *

Please select one answer.

1 = Not at all

2

3

4

5 = To a very great extent

Question 110

To what extent do you think SDCT can be successfully implemented on dairy farms in Romania? *

Please select one answer.

1 = Not at all

2

3

4

5 = To a very great extent

Question 111

What do you consider to be the main obstacle to implementing SDCT? *

Please select one answer.

○ Fear of an increased risk of mastitis

○ Insufficient farmer training

○ Lack of standardized protocols

○ Additional costs

○ Additional workload

○ Other: ______________________

Question 112

What would convince you to recommend or implement SDCT? *(Select all that apply.)* *

☐ Scientific evidence supporting SDCT

☐ Clear implementation protocols

☐ Access to herd data (SCC, bacteriology)

☐ Farmer acceptance

☐ Successful examples from other farms

☐ Reduced costs

☐ Legal requirements/guidelines

☐ If it significantly reduced antibiotic use

☐ Other: ______________________

Question 113

How do you think SDCT would affect farm costs? *

Please select one answer.

○ Costs would increase significantly

○ Costs would increase slightly

○ No impact

○ Costs would decrease

○ I do not know

Question 114

To what extent do you think implementing SDCT could increase the risk of mastitis? *

Please select one answer.

1 = Not at all

2

3

4

5 = To a very great extent

Question 115

To what extent do you think SDCT can be successfully implemented on dairy farms in Romania? *

Please select one answer.

1 = Not at all

2

3

4

5 = To a very great extent

Question 116

To what extent would you be willing to recommend SDCT? *

Please select one answer.

○ Not willing at all

○ Slightly willing

○ Moderately willing

○ Willing

○ Very willing

Question 117

Please provide any comments or concerns regarding the use of SDCT.
